# Novel lncRNA-IUR suppresses Bcr-Abl-induced tumorigenesis through regulation of STAT5-CD71 pathway

**DOI:** 10.1186/s12943-019-1013-3

**Published:** 2019-04-08

**Authors:** Xuefei Wang, Jianling Yang, Guijie Guo, Riyue Feng, Ke Chen, Yuan Liao, Lianfeng Zhang, Liping Sun, Shile Huang, Ji-Long Chen

**Affiliations:** 1 CAS Key Laboratory of Pathogenic Microbiology and Immunology, Institute of Microbiology, Chinese Academy of Sciences (CAS), Beijing, 100101 China; 2 Key Laboratory of Fujian-Taiwan Animal Pathogen Biology, College of Animal Sciences, Fujian Agriculture and Forestry University, Fuzhou, 350002 China; 3 University of Chinese Academy of Sciences, Beijing, China; 4 Department of Immunology, Key Laboratory of Immune Mechanism and Intervention on Serious Disease in Hebei Province, Hebei Medical University, Shijiazhuang, China; 5 Institute of Physical Science and Information Technology, Anhui University, Hefei, 230601 China; 6 Institute of Laboratory Animal Science, Chinese Academy of Medical Sciences & Comparative Medical Center, Peking Union Medical College, Beijing, China; 7 Department of Blood Transfusion, Chinese PLA General Hospital, Beijing, China; 8 Department of Biochemistry and Molecular Biology, Louisiana State University Health Sciences Center, Shreveport, LA USA

**Keywords:** LncRNA, Imatinib, Bcr-Abl, Cellular transformation, Leukemia

## Abstract

**Background:**

Long noncoding RNAs (lncRNAs), defined as the transcripts longer than 200 nt without protein-coding capacity, have been found to be aberrantly expressed in diverse human diseases including cancer. A reciprocal translocation between chromosome 9 and 22 generates the chimeric *Bcr-Abl* oncogene, which is associated with several hematological malignancies. However, the functional relevance between aberrantly expressed lncRNAs and Bcr-Abl-mediated leukemia remains obscure.

**Methods:**

LncRNA cDNA microarray was used to identify novel lncRNAs involved in Bcr-Abl-mediated cellular transformation. To study the functional relevance of novel imatinib-upregulated lncRNA (IUR) family in Abl-induced tumorigenesis, Abl-transformed cell survival and xenografted tumor growth in mice was evaluated. Primary bone marrow transformation and in vivo leukemia transplant using lncRNA-IUR knockdown (KD) transgenic mice were further conducted to corroborate the role of lncRNA-IUR in Abl-induced tumorigenesis. Transcriptome RNA-seq, Western blot, RNA pull down and RNA Immunoprecipitation (RIP) were employed to determine the mechanisms by which lncRNA-IUR-5 regulates Bcr-Abl-mediated tumorigenesis.

**Results:**

We identified a conserved lncRNA-IUR family as a key negative regulator of Bcr-Abl-induced tumorigenesis. Increased expression of lncRNA-IUR was detected in both human and mouse Abl-transformed cells upon imatinib treatment. In contrast, reduced expression of lncRNA-IUR was observed in the peripheral blood lymphocytes derived from Bcr-Abl-positive acute lymphoblastic leukemia (ALL) patients compared to normal subjects. Knockdown of lncRNA-IUR remarkably promoted Abl-transformed leukemic cell survival and xenografted tumor growth in mice, whereas overexpression of lncRNA-IUR had opposite effects. Also, silencing murine lncRNA-IUR promoted Bcr-Abl-mediated primary bone marrow transformation and Abl-transformed leukemia cell survival in vivo. Besides, knockdown of murine lncRNA-IUR in transgenic mice provided a favorable microenvironment for development of Abl-mediated leukemia. Finally, we demonstrated that lncRNA-IUR-5 suppressed Bcr-Abl-mediated tumorigenesis by negatively regulating STAT5-mediated expression of CD71.

**Conclusions:**

The results suggest that lncRNA-IUR may act as a critical tumor suppressor in Bcr-Abl-mediated tumorigenesis by suppressing the STAT5-CD71 pathway. This study provides new insights into functional involvement of lncRNAs in leukemogenesis.

**Electronic supplementary material:**

The online version of this article (10.1186/s12943-019-1013-3) contains supplementary material, which is available to authorized users.

## Background

*Bcr-Abl* oncogene is generated by a reciprocal translocation between chromosome 9 and 22 in human genome, giving Bcr-Abl protein with constitutive tyrosine kinase activity [1]. Bcr-Abl constitutively activates multiple signaling pathways such as Janus family of kinase/signal transducer and activator of transcription (JAK/STAT) pathway, and phosphatidylinositide 3-kinase/protein kinase B (PI3K/AKT) pathway [2–5], which results in cytokine independent proliferation, thereby leading to chronic myeloid leukemia (CML) and acute lymphoblastic leukemia (ALL) [6, 7]. Besides, v-Abl, the retrovirally transduced product of *Abl* gene, contributes to murine pre-B cell malignant transformation [5]. Owing to development of tyrosine kinase inhibitors (TKIs), especially the first-generation imatinib, over 90% of CML patients have been cured in recent years [1, 8, 9]. Imatinib can competitively bind the adenosine triphosphate (ATP) binding pocket of Bcr-Abl, and effectively inhibit its tyrosine kinase activity [8, 9]. Rapidly, the second-generation drugs targeting Bcr-Abl (dasatinib, nilotinib, and bosutinib) and most recently the third-generation inhibitor ponatinib with similar mechanisms have been developed [1]. Although significant progress has been made in treatment of Bcr-Abl-positive hematological malignancies, the precise mechanisms underlying Abl-mediated leukemogenesis are not fully understood.

Human genome transcribes abundant long noncoding RNAs (lncRNAs) that are defined as the transcripts longer than 200 nt without protein-coding capacity. Recently, increasing numbers of lncRNAs have been identified as critical regulators for various biological processes. Dysregulation of lncRNAs is implicated in diverse human diseases [10, 11]. Importantly, numerous lncRNAs are associated with tumorigenesis, such as LINC00312 in lung adenocarcinoma [12], lncRNA-AA174084 in gastric cancer [13], and HOTAIR in multiple types of cancer progression [14]. LncRNA SAMMSON is detectable in over 90% of human melanomas, and silencing SAMMSON drastically decreases the viability of melanoma cells [15]. Moreover, various lncRNAs have been shown as biomarkers or potential targets for diagnosis and treatment of cancer patients [16, 17].

Our previous studies have revealed the functional involvement of lncRNAs in Bcr-Abl-induced CML [18–20]. For example, we have identified lncRNA-BGL3 as a tumor suppressor in Bcr-Abl-mediated tumorigenesis [18]. Upregulation of lncRNA-BGL3 occurs in K562 cells after disruption of Bcr-Abl expression and in primary CML cells derived from patients in response to imatinib treatment; lncRNA-BGL3 functions as a competitive endogenous RNA (ceRNA) to cross-regulate the expression of phosphatase and tensin homolog (PTEN), thereby modulating leukemic cell survival [18]. In addition, we have demonstrated a critical requirement for lncRNA H19 in Bcr-Abl-mediated tumorigenesis [19]. LncRNA H19 expression increases remarkably in Bcr-Abl expressing cell lines and primary CML patients, which is regulated by c-Myc and associated with Bcr-Abl-induced cancer [19]. Despite their importance, the functional relevance of lncRNAs in cellular transformation by *Abl* oncogenes remains largely unknown.

This study was set to comprehensively evaluate the expression of lncRNAs in human K562 leukemic cells in response to imatinib treatment. Using lncRNA cDNA microarray, we identified a conserved, imatinib-upregulated lncRNA family, which negatively regulates Bcr-Abl-mediated cell survival and tumorigenesis by suppressing the STAT5-CD71 pathway, as demonstrated by both in vitro and in vivo experiments. The findings advance our understanding of complicated mechanisms underlying Bcr-Abl-induced hematopoietic malignancies.

## Materials and methods

### Ethics statement

All animal experiments were approved by the Research Ethics Committee of Institute of Microbiology, Chinese Academy of Sciences (Permit Number: PZIMCAS2013008). All mouse experimental procedures were performed in accordance with the Regulations for the Administration of Affairs Concerning Experimental Animals approved by the State Council of People’s Republic of China. All participants signed informed consent prior to using the peripheral blood cells for scientific research.

### Microarray and RNA-seq analysis

The lncRNA cDNA microarray was from Agilent (Santa Clara, CA, USA). Total RNAs from three independent groups of K562 cells treated with imatinib or mock were prepared using Trizol reagent (Invitrogen, Carlsbad, CA, USA). The sample labeling, hybridization and data analysis were performed as previously described [18]. The microarray data have been deposited in the NCBI Gene Expression Omnibus (http://www.ncbi.nlm.nih.gov/geo/ accession number GSE119770).

Total RNAs used for RNA-seq were isolated from three independent groups of K562 cell lines expressing short hairpin RNA (shRNA) targeting lncRNA-IUR-5 and control cells, using TRIzol reagent (Invitrogen). RNA libraries and data procession were described in Additional file 1: Supplementary Materials and Methods. These RNA-seq data have been deposited on GEO public database under the accession number GSE120337.

### Cell lines, cell culture, and clinical samples

Cell lines K562, 293 T and Sup-B15 were purchased from American Type Culture Collection (Manassas, VA, USA). Huh7 and MCF-7 were purchased from National Platform of Experimental Cell Resources for Sci-Tech (http://cellresource.cn, Beijing, China). The v-Abl-transformed mouse cell lines NS2 and W44 were generated and cultured as described previously [3]. Cells were grown in Dulbecco’s modified Eagle medium (DMEM) or RPMI 1640 supplemented with 10% fetal bovine serum and antibiotics (penicillin and streptomycin) as previously described [2, 18]. Peripheral blood lymphocytes derived from Bcr-Abl-positive ALL patients and normal people were collected from the Department of Blood Transfusion, Chinese PLA General Hospital, Beijing, China by Liping Sun. The basic information of these clinical samples was listed in Table 1.

**Table 1:** Basic information of Bcr-Abl-positive ALL patients and normal control

	Number	Age (years)	Gender (Male/Female)
Normal people	1	39	male
	2	23	male
	3	56	male
	4	31	female
	5	20	male
ALL patients	1	4	female
	2	5	female
	3	44	male
	4	32	male
	5	18	male

### Flow cytometry and apoptosis assay

Apoptosis assay was performed as previously described [18]. Briefly, cells were treated with imatinib (10 μM) for indicated time, then stained with propidium iodide (PI)/Annexin V and analyzed by fluorescence activated cell sorter (FACS) (BD Biosciences, San Jose, CA, USA).

### Nude mice injection

Nude mice injection was carried out as previously described [18]. Bioluminescent imaging was performed to probe tumor growth from GFP-expressing cells.

### Primary murine bone marrow transformation assay

Primary bone marrow transformation was performed as previously described [2, 18]. Transformation efficiency was measured by counting the number of Bcr-Abl-transformed cell clones.

### Transgenic mice and in vivo leukemia transplant

The lncRNA-IUR-knockdown transgenic mice were generated using sh-IUR-m45678 by the microinjection method as previously described [18]. The transgenic founders with high interference efficiency were selected and maintained on a C57BL/6J genetic background. For leukemia transplantation, GFP-positive NS2 cells (1 × 10^7^) were injected into sub-lethally (5.5 Gy, X-ray) irradiated recipients (C57BL/6 mice) through tail vein.

### 5′ and 3′ RACE

The 5′ and 3′ RACE analyses were performed using the SMARTer RACE cDNA amplification Kit (Clontech, Mountain View, CA, USA) [21]. RACE PCR products were cloned into pZeroBack (Tiangen, Beijing, China) and sequenced.

### RNA pull-down assay and RNA immunoprecipitation

K562 cell lines overexpressing S1, lncRNA-IUR-5, lncRNA-IUR-5-S1 were generated and used for RNA pull-down assay as described in Additional file 1: Supplementary Materials and Methods [22, 23]. K562 cells upon imatinib treatment were subjected to RIP using Magna RIP™ RNA-Binding Protein Immunoprecipitation Kit (Millipore, Burlington, MA, USA) [21]. Rabbit anti-STAT5 (3H7) antibody (Cell Signaling, Danvers, MA, USA, #9358) was used in this experiment.

### Statistical analysis

Statistical significance was determined by Student’s *t*-test. Data represent the mean ± SEM and *p* values < 0.05 was considered to be significant.

## Results

### LncRNA-IUR family is identified as novel lncRNAs in leukemic cells induced by imatinib treatment

**Fig. 1:**
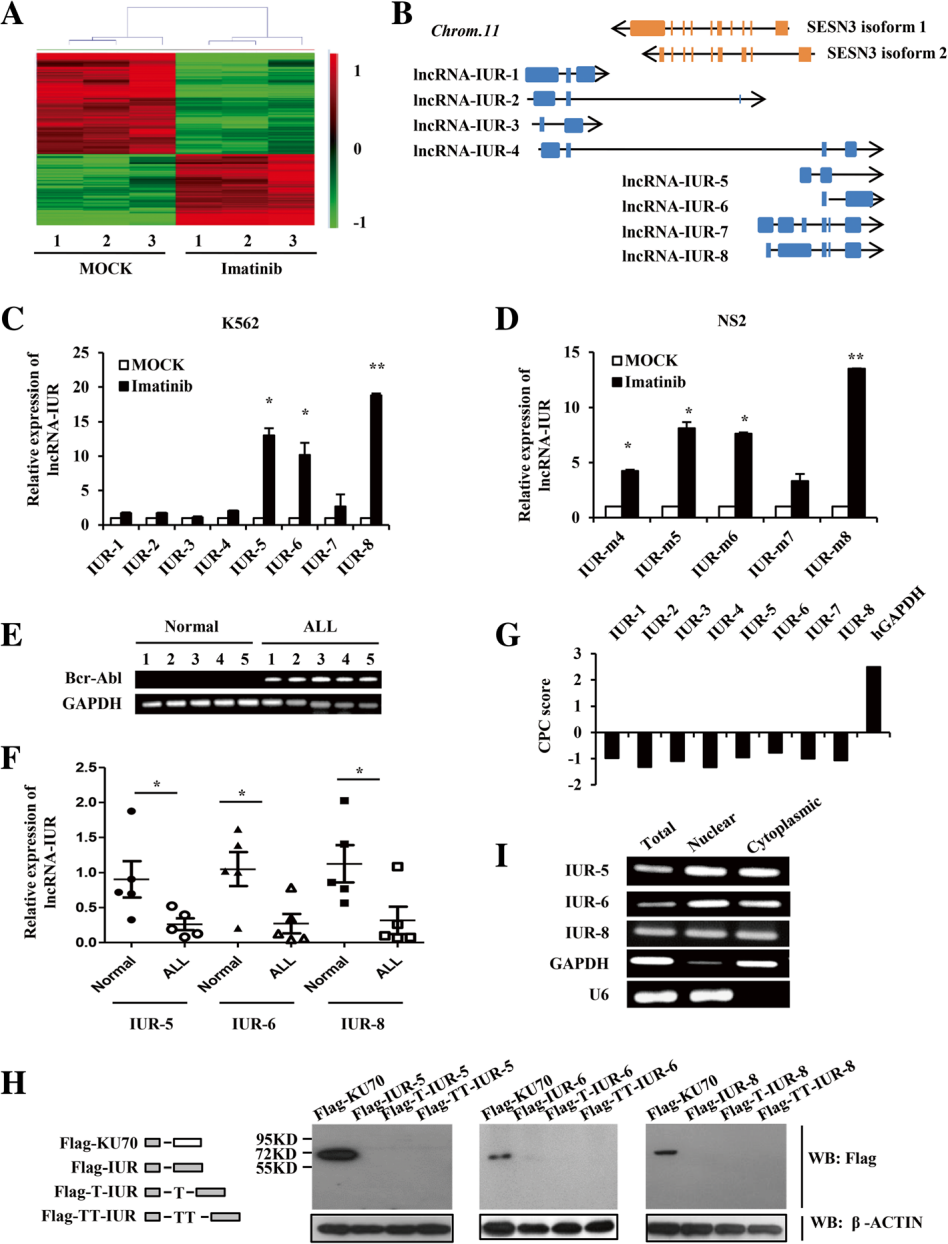
Identification of the novel imatinib-upregulated lncRNA family. **a**, The hierarchical clustering analysis of lncRNAs differentially expressed in K562 cells treated with or without imatinib (n = 3; fold change > 2.0; *p* < 0.05). The values from three independent experiments were displayed. The microarray analysis revealed 987 upregulated and 1479 downregulated lncRNAs upon treatment with imatinib. **b**, The paradigm of the human genomic location of lncRNA-IUR family (blue) and SESN3 (orange). The orientation of arrows indicated the transcription direction. **c** and **d**, Quantitative real-time PCR analysis of human lncRNA-IUR expression in K562 cells (**c**) and murine lncRNA-IUR expression in NS2 cells (**d**) in response to treatment with imatinib (n = 3; means ± SEM; **p < 0.01; *p < 0.05). **e**, RT-PCR analysis of Bcr-Abl expression in the primary peripheral blood lymphocytes from Bcr-Abl-positive ALL patients and normal subjects. **f**, Quantitative real-time PCR analysis of lncRNA-IUR expression in primary leukemic cells from Bcr-Abl-positive ALL patients and normal control (n = 5; means ± SEM; *p < 0.05). **g**, The CPC scores for each transcript of lncRNA-IUR family and control gene GAPDH (http://cpc.cbi.pku.edu.cn). **h**, LncRNA-IUR-5, 6, and 8 (full length) were cloned into pNL vector with N-terminal Flag tag in all three reading frames. The plasmids were transfected into 293 T cells for 48 h. Cell lysates were harvested and subjected to Western blotting (WB) with anti-Flag. Flag-KU70 served as a positive control. **i**, RT-PCR was performed to examine cytoplasmic or nuclear lncRNA-IUR expression in K562 cells. GAPDH served as a cytoplasmic control, and U6 as a nuclear control

To identify novel lncRNAs involved in Bcr-Abl-mediated cellular transformation, an lncRNA cDNA microarray (Agilent) was used to comprehensively analyze the expression of lncRNAs in K562 cells in response to treatment with imatinib (Abl kinase inhibitor). Numerous lncRNAs were found to be differentially expressed in K562 cells exposed to imatinib (Fig. 1a). Of particular interest, the imatinib-treated cells had a significantly increased expression of several transcripts of an lncRNA family that we call imatinib-upregulated lncRNA (lncRNA-IUR). There were 8 members (transcripts) in this family in human cells (Fig. 1b and Additional file 2: Figure S1A) and 5 transcripts were homologous in mouse cells (Additional file 2: Figure S1B). The upregulation of lncRNA-IUR by imatinib was further confirmed in the leukemic cells by quantitative real-time PCR (Fig. 1c). In particular, transcripts 5, 6, and 8 of lncRNA-IUR family were most prominently induced in K562 cells by imatinib treatment (Fig. 1c). Similar results were observed in v-Abl-transformed murine cell lines NS2 and W44 (Fig. 1d and Additional file 2: Figure S1C). Moreover, transcripts 1, 4 and 5 of lncRNA-IUR family were also upregulated in another Bcr-Abl-positive cell line (Sup-B15) after imatinib treatment (Additional file 2: Figure S1D).

Next, we examined the expression of lncRNA-IUR in clinical samples. The results indicated that the level of lncRNA-IUR in the peripheral blood lymphocytes derived from Bcr-Abl-positive ALL patients was significantly lower than that in normal subjects (Fig. 1e and f, and Additional file 2: Figure S1E). Together, the above findings from both leukemic cell lines and clinical samples indicate that the expression of lncRNA-IUR is suppressed in Abl-transformed leukemic cells, but is induced by imatinib treatment.

Furthermore, we analyzed the coding potential of lncRNA-IUR through software prediction and experiments. Using the Open Reading Frame (ORF) Finder, we found that the potential ORFs for each transcript of lncRNA-IUR family were totally shorter than 300 bp (Additional file 2: Figure S1F). There were also no matched peptides predictably translated from lncRNA-IUR by comparing in UniProtKB/Swiss-Prot (swissprot) database. Furthermore, the Coding Potential Calculator (CPC) was also employed to assess the coding potential of lncRNA-IUR family. The CPC score for each transcript was minus, indicating “noncoding” (Fig. 1g). Using in vitro translation experiments, we were able to observe a specific protein band (about 70 kD) for the control gene KU70, but failed to detect any protein bands for lncRNA-IUR-5, 6 or 8 (Fig. 1h), supporting that lncRNA-IUR had no coding potential. In addition, a full length of lncRNA-IUR-5 (totally 2342 nt) was identified by RACE experiments and submitted to GenBank (MH899175) (Additional file 2: Figure S1G and S1H). We also showed that lncRNA-IUR-5, 6 and 8 were localized in both cytoplasm and nucleus by cellular fractionation experiments (Fig. 1i).

### Altering lncRNA-IUR expression affects Abl-transformed cell survival in vitro and tumor growth in vivo

**Fig. 2:**
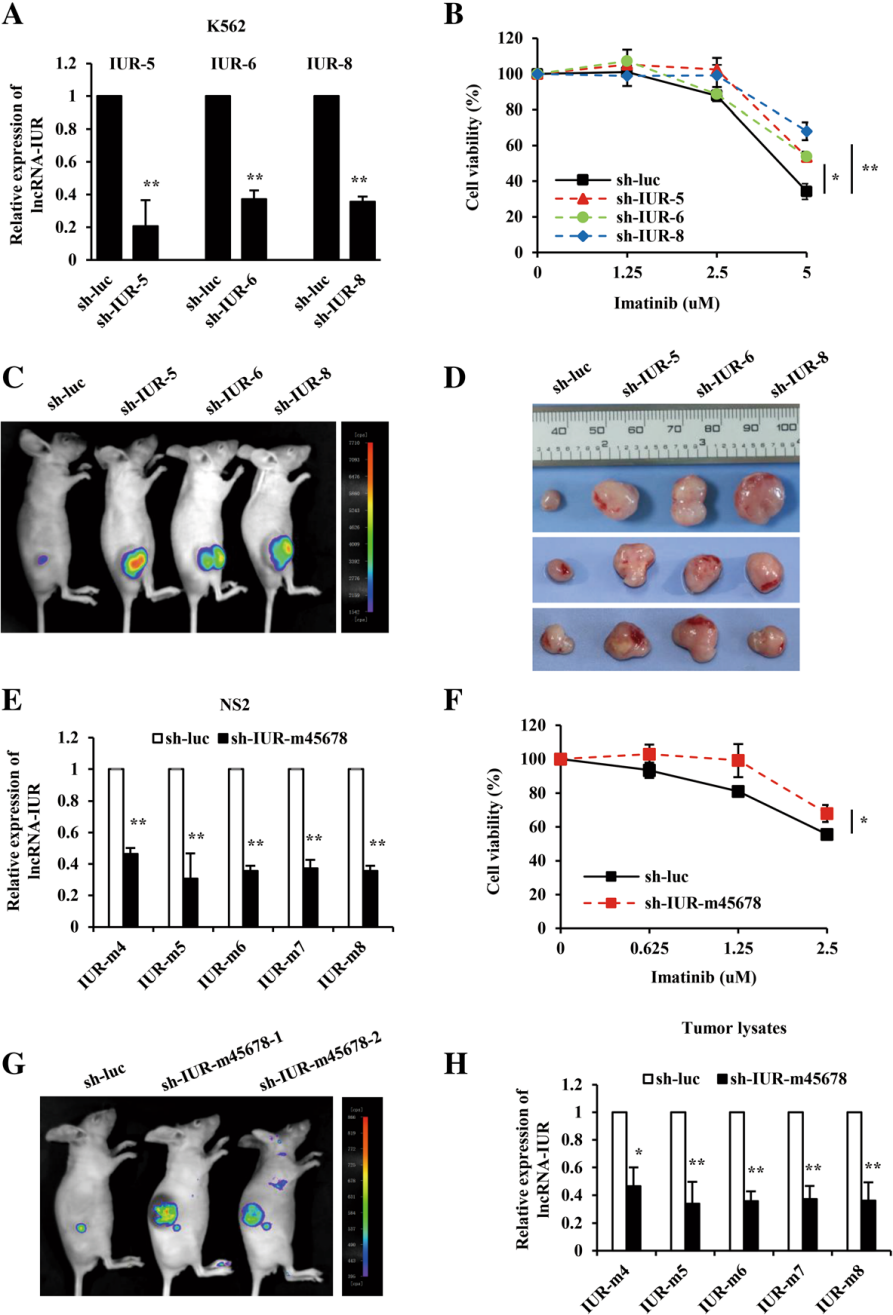
Depletion of lncRNA-IUR expression enhances Abl-transformed cell survival and tumor growth in a xenograft mouse model. **a**, Quantitative real-time PCR was performed to examine lncRNA-IUR expression in K562 cell lines stably expressing sh-IUR-5, -6, -8 or sh-luc (n = 3; means ± SEM; **p < 0.01). **b**, Cell viability of indicated K562 cell lines was analyzed by flow cytometry upon treatment with indicated concentrations of imatinib for 36 h (n = 3; means ± SEM; **p < 0.01; *p < 0.05). **c** and **d**, Nude mice were subcutaneously injected with indicated K562 cell lines. Tumor growth was measured by bioluminescent imaging (**c**), and then excised from the nude mice (**d**). Shown were representative images from at least three independent experiments with similar results. **e**, Quantitative real-time PCR was performed to examine the murine lncRNA-IUR expression in NS2 cell lines expressing sh-IUR-m45678 or sh-luc (n = 3; means ± SEM; **p < 0.01). **f**, Cell viability of indicated NS2 cell lines was analyzed by flow cytometry after treatment with indicated concentrations of imatinib for 36 h (n = 3; means ± SEM; *p < 0.05). **g** and **h**, Nude mice were subcutaneously injected with indicated NS2 cell lines. Tumor growth was measured by bioluminescent imaging (**g**), and analysis of murine lncRNA-IUR expression in these tumor lysates was measured by quantitative real-time PCR (n = 3; means ± SEM; **p < 0.01; *p < 0.05) (**h**)

To understand the functional significance of lncRNA-IUR family in Abl-transformed leukemic cells, we investigated whether altering lncRNA-IUR expression has any effect on cell survival and tumor formation in a xenograft mouse model. For this, stable K562 cell lines were generated using shRNAs specifically targeting transcripts 5, 6, and 8 of lncRNA-IUR (sh-IUR-5, -6, and -8) or luciferase control (sh-luc) (Fig. 2a). We observed that knocking down lncRNA-IUR-5 did not affect the expression of lncRNA-IUR-6/8 (Additional file 2: Figure S2A). Similarly, knocking down lncRNA-IUR-6 or lncRNA-IUR-8 did not either influence the expression of lncRNA-IUR-5/8 or lncRNA-IUR-5/6 (Additional file 2: Figure S2A). The results suggest that there may be no interference between the transcripts of these lncRNA-IUR family members each other. Next, we evaluated their functional significance in Abl-transformed leukemic cells independently. As shown in Fig. 2b, the viability of lncRNA-IUR knockdown K562 cells was significantly higher than that of control group after imatinib treatment. However, no significant difference in cell cycle progression was observed between the lncRNA-IUR knockdown and control cells (Additional file 2: Figure S2B). Furthermore, we employed a xenograft mouse model in which each mouse was inoculated subcutaneously with K562 cells expressing shRNAs targeting either lncRNA-IUR or luciferase control. Knockdown of lncRNA-IUR 5, 6 or 8 strikingly promoted the tumor growth (Fig. 2c and d). Downregulation of lncRNA-IUR in tumors was further confirmed by quantitative real-time PCR analysis (Additional file 2: Figure S2C).

In addition, v-Abl-transformed NS2 cell line was used and 5 transcripts of murine lncRNA-IUR were knocked down using sh-IUR-m45678 (Fig. 2e). Consistently, we found that depletion of murine lncRNA-IUR in NS2 cells promoted cell survival (Fig. 2f). Furthermore, tumors formed by NS2 cells expressing sh-IUR-m45678 were much larger than those formed by control cells (Fig. 2g). Downregulation of lncRNA-IUR in the tumors was also confirmed by quantitative real-time PCR analysis (Fig. 2h). Collectively, these results indicate that downregulation of lncRNA-IUR enhances Abl-transformed leukemic cell survival in culture and tumor growth in a xenograft mouse model.

**Fig. 3:**
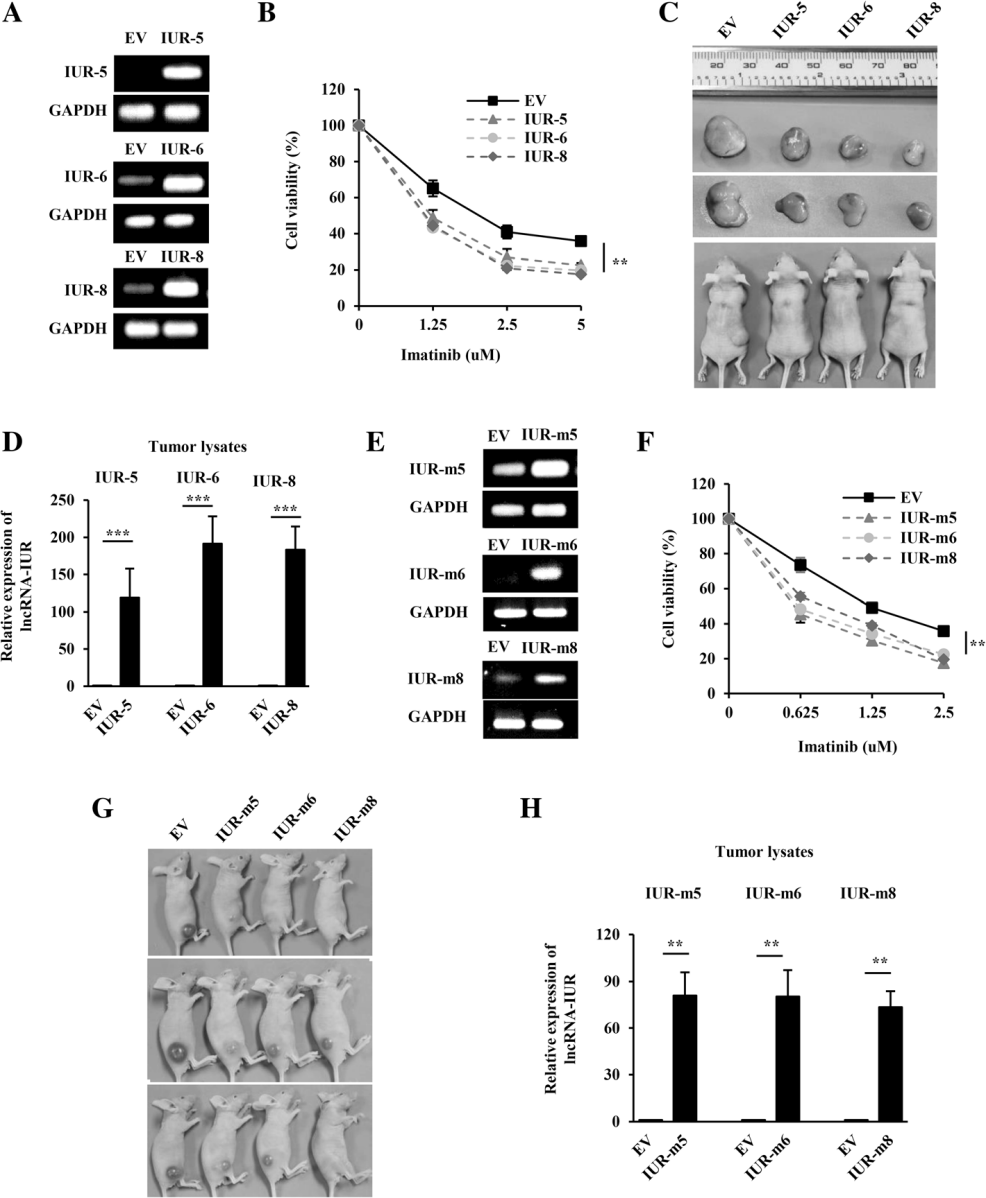
LncRNA-IUR overexpression sensitizes Abl transformant to undergo apoptosis and attenuates tumor growth in a xenograft mouse model*.* **a**, RT-PCR was performed to examine lncRNA-IUR expression in K562 cell lines overexpressing lncRNA-IUR-5, -6, -8 or empty vector (EV). **b**, Cell viability of indicated K562 cell lines was analyzed by flow cytometry after treatment with indicated concentrations of imatinib for 36 h (n = 3; means ± SEM; **p < 0.01). **c**, Nude mice were subcutaneously injected with indicated K562 cell lines. Shown were representative images of tumors excised from these nude mice. **d**, Analysis of lncRNA-IUR expression in indicated tumor lysates by quantitative real-time PCR (n = 3; means ± SEM; ***p < 0.001). **e**, RT-PCR was performed to examine the murine lncRNA-IUR expression in NS2 cell lines overexpressing lncRNA-IUR-m5, -m6, -m8 or EV. **f**, Cell viability of indicated NS2 cell lines was analyzed by flow cytometry after treatment with indicated concentrations of imatinib for 36 h (n = 3; means ± SEM; **p < 0.01). **g**, Shown were representative images of nude mice subcutaneously injected with indicated NS2 cell lines. **h**, Murine lncRNA-IUR expression in these tumor lysates was analyzed by quantitative real-time PCR (n = 3; means ± SEM; **p < 0.01)

On the other hand, we also tested the effect of lncRNA-IUR overexpression on cell survival and tumorigenesis. To this end, K562 cell lines overexpressing lncRNA-IUR transcript 5, 6, or 8 (IUR-5, -6, and -8) and empty vector (EV) were generated (Fig. 3a and Additional file 2: Figure S2D). We found that overexpression of lncRNA-IUR sensitized K562 cells to undergo imatinib-induced apoptosis (Fig. 3b). Also, as expected, overexpression of lncRNA-IUR profoundly suppressed the growth of K562 xenografts in nude mice (Fig. 3c). Overexpression of lncRNA-IUR in tumors was confirmed by quantitative real-time PCR analysis (Fig. 3d). Similar results were obtained using NS2 cell lines overexpressing either murine lncRNA-IUR transcript 5, 6, 8 (IUR-m5, -m6, and -m8) or empty vector (EV) (Fig. 3e-h and Additional file 2: Figure S2E). These experiments demonstrated that lncRNA-IUR overexpression impaired Abl-transformed leukemic cell survival and suppressed tumor growth. The data support the notion that lncRNA-IUR family members, especially transcripts 5, 6 and 8, may act as critical tumor suppressors during Abl-induced tumorigenesis.

### Silencing murine lncRNA-IUR promotes Abl-mediated bone marrow cell transformation and leukemic cell growth in vivo

**Fig. 4:**
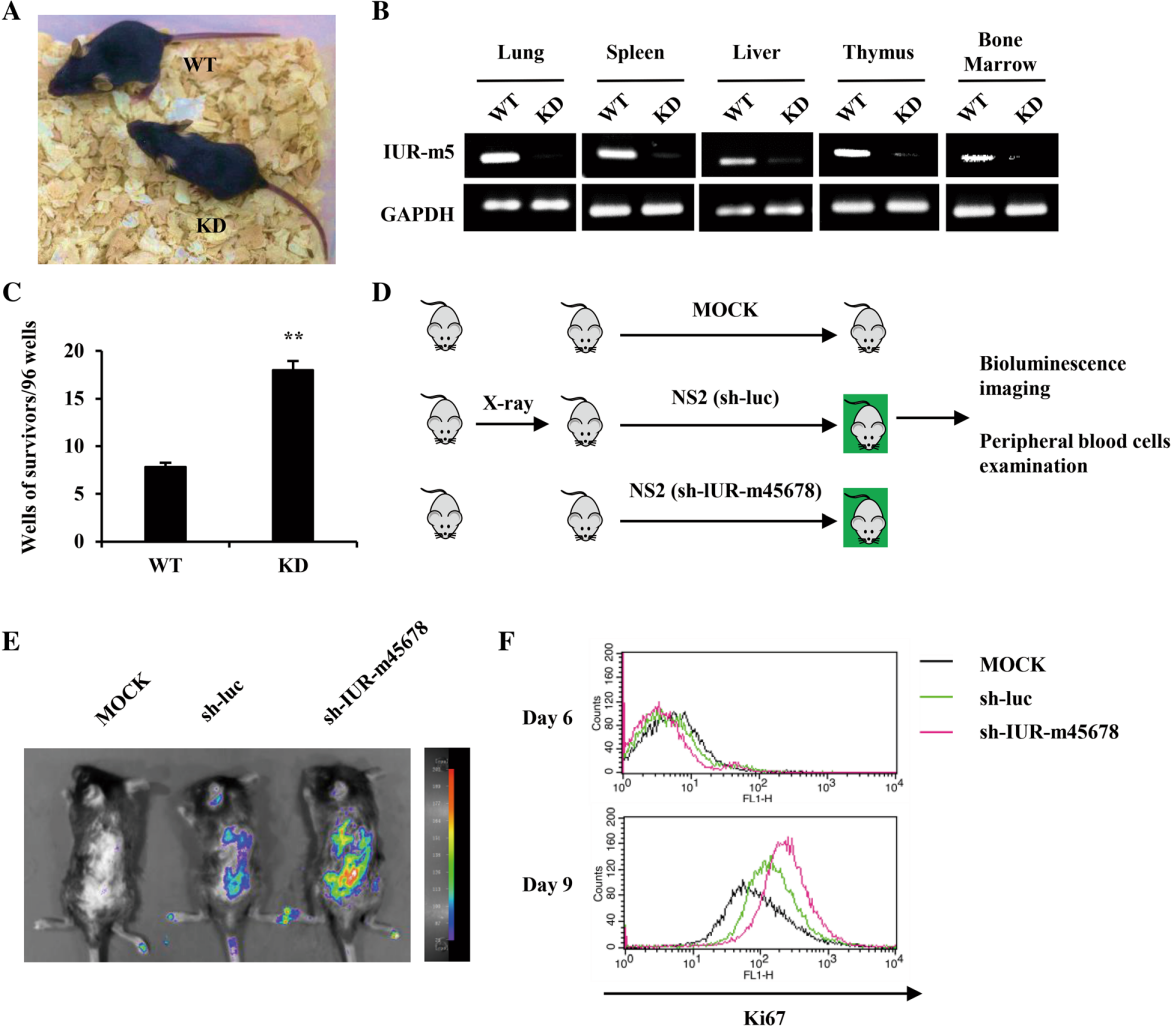
Silencing murine lncRNA-IUR promotes Abl-mediated bone marrow cell (BMC) transformation and NS2 cell growth in vivo. **a**, The photo of lncRNA-IUR KD transgenic mice and their WT littermate. **b**, RT-PCR was performed to examine the lncRNA-IUR-m5 expression in multiple organs of lncRNA-IUR KD mice and WT mice. **c**, Survived clone number of Bcr-Abl-transformed BMCs from WT and KD mice were scored as described in Materials and Methods (n = 5; means ± SEM; **p < 0.01). **d**, Scheme of in vivo leukemia transplant into WT mice infusing GFP-positive NS2 cells expressing sh-IUR-m45678, sh-luc or equal volume of PBS. Three groups were labeled as sh-IUR-m45678, sh-luc, and MOCK. **e**, Bioluminescent imaging shown distribution of GFP-positive NS2 cells stably expressing sh-IUR-m45678 or sh-luc in WT mice at the 8th day after in vivo leukemia transplantation. MOCK group served as a negative control. Shown were representative images from at least three independent experiments with similar results. **f**, Ki67 expression in PBCs of WT mice at the 6th and 9th day after in vivo leukemia transplantation as indicated was examined by flow cytometry

To further define the inhibitory role of lncRNA-IUR family in Abl-mediated tumorigenesis, lncRNA-IUR knockdown (KD) transgenic mice were generated using sh-IUR-m45678 (Fig. 4a). Using both RT-PCR and quantitative real-time PCR, a potent knockdown efficiency was detected in multiple organs of the KD mice, including lung, spleen, liver, thymus and bone marrow (Fig. 4b and Additional file 2: Figure S3A). Primary bone marrow transformation was performed by using primary bone marrow cells (BMCs) derived from lncRNA-IUR KD transgenic mice or their wild-type (WT) littermates, and then infecting these BMCs with the retrovirus carrying *Bcr-Abl* oncogene. Transformation efficiency was measured by counting the clone number of Bcr-Abl-transformed BMCs. As shown in Fig. 4c, the transformation efficiency from the KD mice was significantly higher than that from the wild-type counterparts. The results indicate that disrupting murine lncRNA-IUR expression promotes Bcr-Abl-mediated primary bone marrow transformation.

Next, we examined the effect of lncRNA-IUR deficiency on Abl-transformed leukemic cell growth in vivo. A leukemia mouse model was generated by injecting sub-lethally irradiated WT mice with GFP-positive NS2 cells stably expressing sh-IUR-m45678 or sh-luc, or equal volume of mock as negative control (Fig. 4d). Under the intensity of X-ray irradiation we employed, the number of white blood cells (WBCs) from C57BL/6J genetic background mice severely decreased and resulted in immunity injury (Additional file 2: Figure S3B). Bioluminescence imaging showed that the intensity of GFP signal in mice injected with sh-m45678-expressing NS2 cells was much stronger than that in other control groups (Fig. 4e). Analysis of proliferation marker Ki67 in peripheral blood cells (PBCs) by flow cytometry revealed that the level of Ki67 was greatly elevated by silencing lncRNA-IUR in sh-IUR-m45678 group (Fig. 4f). In addition, spleen from mice injected with sh-IUR-m45678-expressing NS2 cells displayed clear splenomegaly as compared with other groups (Additional file 2: Figure S3C). These results suggest that silencing murine lncRNA-IUR expression promotes Abl transformant tumor growth in vivo.

### Silencing murine lncRNA-IUR in transgenic mice provides an ideal microenvironment for Abl-mediated leukemia development

**Fig. 5:**
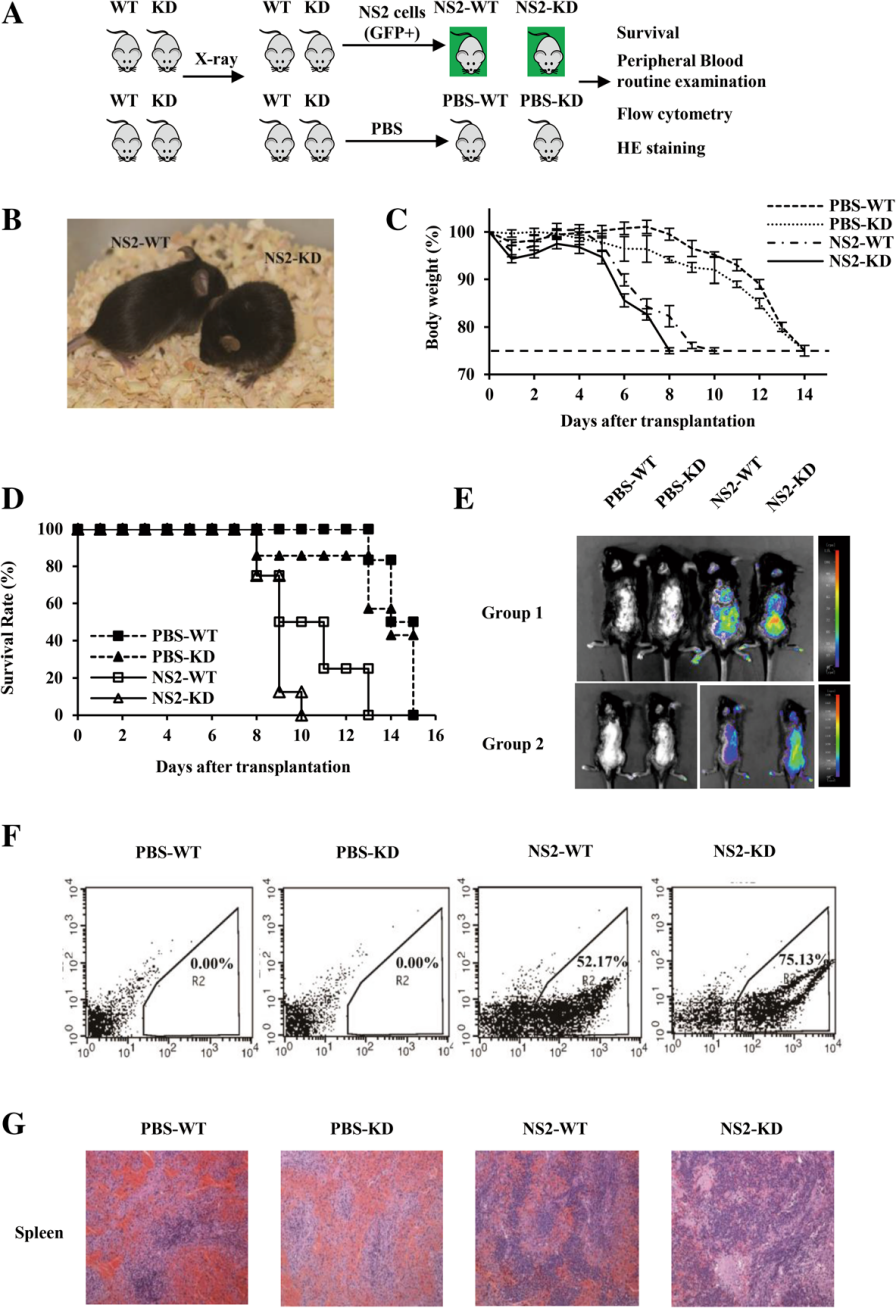
LncRNA-IUR knockdown transgenic mice provides an ideal microenvironment for Abl-mediated leukemia development. **a**, Scheme of in vivo leukemia transplant using KD and WT mice. Briefly, lncRNA-IUR KD mice and their WT littermates were infused with GFP-positive NS2 cells or PBS by vena caudalis after sub-lethally irradiation. Experimental groups were labeled as NS2-KD and NS2-WT, and control groups were labeled as PBS-KD and PBS-WT. **b**, Representative photo shown NS2-KD and NS2-WT mice at the 8th day after in vivo leukemia transplantation. **c** and **d**, Body weight (**c**) and survival rate (**d**) of indicated mice. Each group contained 8-10 mice. Mice were monitored for a period of 15 days. **e**, Bioluminescent imaging was performed to examine distribution of GFP-positive NS2 cells in indicated mice. **f**, Percentage of GFP-positive NS2 cells in PBCs of indicated mice was detected by flow cytometry. Shown were representative images from at least three independent experiments. **g**, Shown were representative images of spleen from indicated mice by HE staining

To further address the functional relevance of lncRNA-IUR in Abl-mediated tumorigenesis, we used the lncRNA-IUR KD transgenic mice and WT littermates to develop leukemia. The KD mice and WT littermates were sub-lethally irradiated and infused with GFP-positive v-Abl-transformed NS2 cells through vena caudalis (Fig. 5a). Experimental groups injected with NS2 cells were labeled as NS2-KD and NS2-WT, and mock treated control groups were labeled as PBS-KD and PBS-WT. The incidence of leukemia in the KD mice was 100%, having no natural relief. Interestingly, almost all the NS2-KD mice developed signs of the disease within 8 days post injection, whereas the majority of NS2-WT group and control groups remained normal under the same condition (Fig. 5b and Additional file 2: Figure S3D). The body weight of the NS2-KD group lost much faster than that of the NS2-WT group (Fig. 5c), and all NS2-KD mice died within 10 days (Fig. 5d). Besides, the number of WBCs in the peripheral blood increased significantly in the NS2-KD group compared to that in the NS2-WT group (Additional file 2: Figure S3E). However, the number of red blood cells (RBCs) and platelets (PLT) in the peripheral blood did not change significantly during the course of the experiment (Additional file 2: Figure S3F and S3G). These data indicate that mice with deficient lncRNA-IUR are highly susceptible to develop leukemia.

In addition, we observed that the intensity of GFP signal was much stronger in the whole body of the NS2-KD group than the NS2-WT group through bioluminescence imaging (Fig. 5e). Consistently, the proportion of GFP-positive NS2 cells in PBCs of the KD group was much higher than that in the WT group by analysis of flow cytometry (Fig. 5f and Additional file 2: Figure S3H). The results suggest that it is much easier for NS2 cells to survive and grow in lncRNA-IUR KD mice compared to WT mice. Additionally, compared to PBS-WT or PBS-KD group, both NS2-KD and NS2-WT groups displayed splenomegaly (Additional file 2: Figure S3I). There was no significant difference in the spleen weight between NS2-KD and NS2-WT groups (Additional file 2: Figure S3J), but hematoxylin and eosin (HE) staining and bioluminescence imaging revealed that more leukemic cells were dispersedly distributed in the spleens of the NS2-KD group (Fig. 5g and Additional file 2: Figure S3K). These data indicate that NS2 cells are more inclined to infiltrate into the spleen of the lncRNA-IUR KD mice than that of WT mice after induction of leukemia. Together, these observations suggest that knockdown of lncRNA-IUR in mice provides an ideal tumor microenvironment for Abl-mediated leukemia development.

### LncRNA-IUR-5 negatively regulates STAT5-mediated CD71 expression

**Fig. 6:**
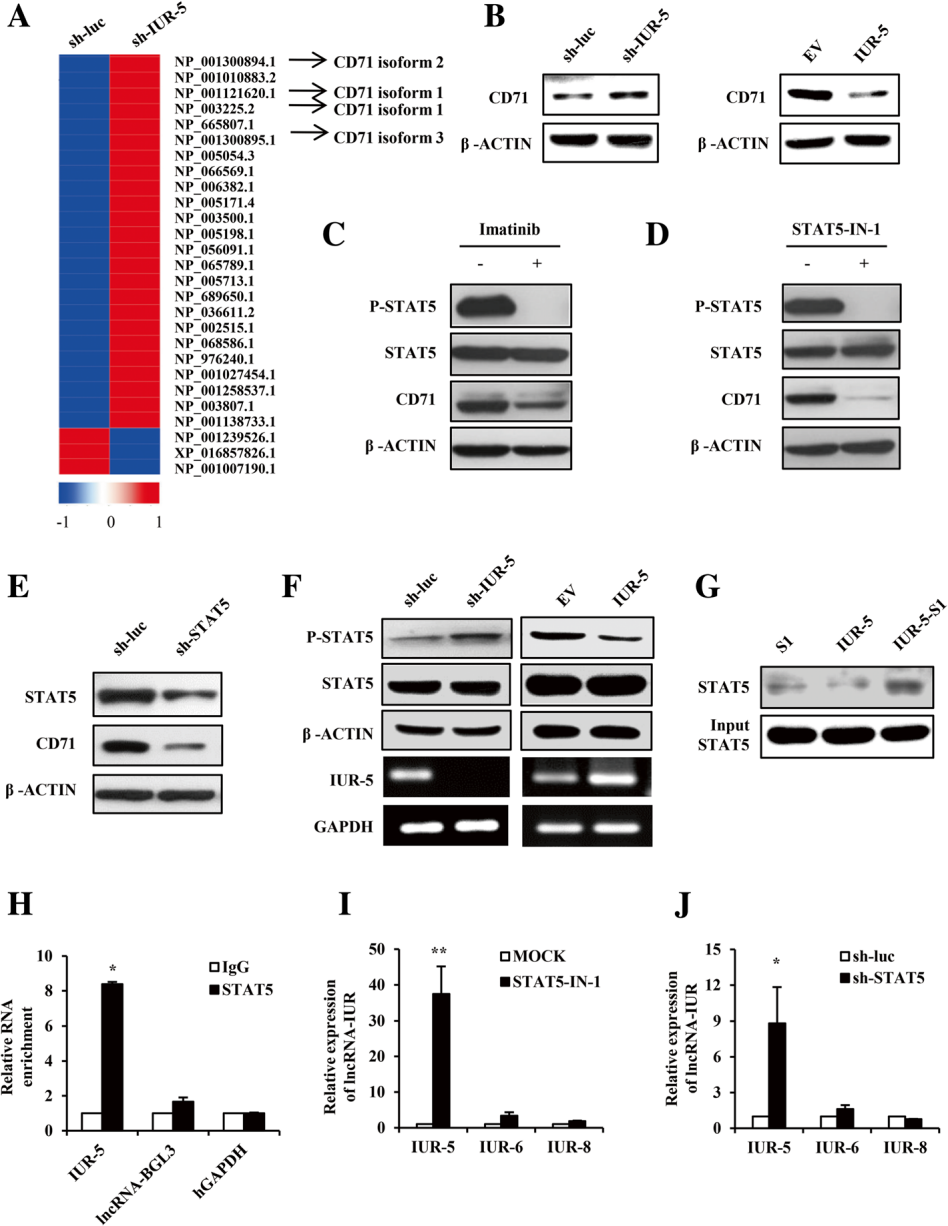
LncRNA-IUR-5 regulates STAT5-mediated CD71 expression. **a**, Transcriptome RNA sequencing analysis of lncRNA-IUR-5 knockdown K562 cell lines and control cells. Arrows indicated three isoforms of CD71. **b**, Western blotting analysis of CD71 levels in lncRNA-IUR-5 knockdown or overexpressing K562 cell lines. **c-e**, Western blotting analysis of CD71 levels in K562 cells upon imatinib treatment (**c**), STAT5-IN-1 (50 μM, 6 h) treatment (**d**), or knocking down STAT5 using sh-STAT5 (**e**). **f**, Western blotting and RT-PCR were performed to examine the protein and mRNA levels as indicated. **g**, Western blotting analysis of purified binding proteins with lncRNA-IUR-5, which were pulled down from K562 cell lines stably expressing S1, IUR-5 or IUR-5-S1. **h**, The interaction between lncRNA-IUR-5 and STAT5 was verified through RIP. lncRNA-BGL3 and GAPDH served as negative controls (n = 3; means ± SEM; *p < 0.05). **i** and **j**, Quantitative real-time PCR was performed to examine lncRNA-IUR expression in K562 cells upon STAT5-IN-1 (50 μM, 6 h) treatment (**i**) or knocking down STAT5 using sh-STAT5 (**j**) (n = 3; means ± SEM; **p < 0.01; *p < 0.05)

Next, we sought to determine the mechanism of the tumor suppressive action of lncRNA-IUR. Since our lncRNA microarray data showed that lncRNA-IUR-5 expression was significantly increased upon imatinib treatment (Additional file 2: Figure S4A), we hypothesized that lncRNA-IUR-5 may target the Bcr-Abl-mediated expression of genes. To test this hypothesis, we performed transcriptome RNA-seq analysis of lncRNA-IUR-5 knockdown K562 cells and control cells (Fig. 6a). Disruption of lncRNA-IUR-5 significantly altered the expression of 39 genes. Interestingly, all three isoforms of transferrin receptor protein (TfR or CD71) were greatly upregulated in lncRNA-IUR-5 knockdown cells (Fig. 6a). CD71 is a cell membrane-associated glycoprotein involved in the cellular uptake of iron and the regulation of cell growth [24], and CD71 is ubiquitously expressed at a low level in most normal human tissues but greatly elevated in cancer cells, which makes it an attractive target for cancer therapy including leukemia [25–27]. We confirmed that protein expression of CD71 was increased after silencing lncRNA-IUR-5, whereas its level was reduced by overexpressing lncRNA-IUR-5 in K562 cells (Fig. 6b). These data indicate that lncRNA-IUR-5 negatively regulates the expression of CD71 in the leukemic cells.

STAT5 has been reported as a transcription factor of CD71 [28]. Importantly, STAT5 is a key factor implicated in numerous human cancers [29–31]. Activation of STAT5 is critically associated with Abl-mediated leukemia and can be effectively inhibited by imatinib [32–34]. In this study, we found that the phosphorylation level of STAT5 and protein level of CD71 were markedly reduced in K562 cells treated with imatinib (Fig. 6c). Furthermore, inhibition of STAT5 activity by STAT5-IN-1, an inhibitor of STAT5, caused a striking decrease in the protein level of CD71 (Fig. 6d). Similar results were obtained from experiments using shRNA specifically targeting STAT5 in K562 cells (Fig. 6e), confirming that STAT5 positively regulates CD71 expression in the Bcr-Abl expressing leukemic cells.

Next, we further checked whether the inhibitory effect of lncRNA-IUR-5 on CD71 expression is dependent on STAT5 signaling. As predicted, depletion of lncRNA-IUR-5 expression increased STAT5 phosphorylation, whereas forced expression of lncRNA-IUR-5 reduced STAT5 phosphorylation (Fig. 6f). To further identify potential binding protein(s) of lncRNA-IUR-5, we carried out RNA pull-down experiment by using an S1 aptamer-tagged lncRNA-IUR-5 (IUR-5-S1, untagged IUR-5 or S1 tag as negative controls) as previously described [22]. Interestingly, STAT5A and STAT5B were found among the proteins identified by mass spectrometry of the RNA pull-down (Additional file 2: Figure S4B and S4C). This finding was confirmed by independent Western blotting (Fig. 6g). Subsequently, RIP further verified the specificity of the interaction between lncRNA-IUR-5 and STAT5 (Fig. 6h). These results suggest that lncRNA-IUR-5 interacts with STAT5 and inhibits its activation.

On the other hand, we also tested whether STAT5 was involved in regulating lncRNA-IUR expression. Treatment of K562 cells with STAT5-IN-1 caused a significant increase in lncRNA-IUR-5 level but not lncRNA-IUR-6 and lncRNA-IUR-8 (Fig. 6i and Additional file 2: Figure S4D). Similar results were obtained from experiments using shRNA to target STAT5 in K562 cells (Fig. 6j and Additional file 2: Figure S4E). Taken together, the above results suggest that there is a reciprocal regulation between lncRNA-IUR-5 and STAT5, and activation of STAT5 leads to suppression of lncRNA-IUR-5 expression in the leukemic cells.

### LncRNA-IUR-5 inhibits Abl-mediated tumorigenesis by suppressing CD71 expression

**Fig. 7:**
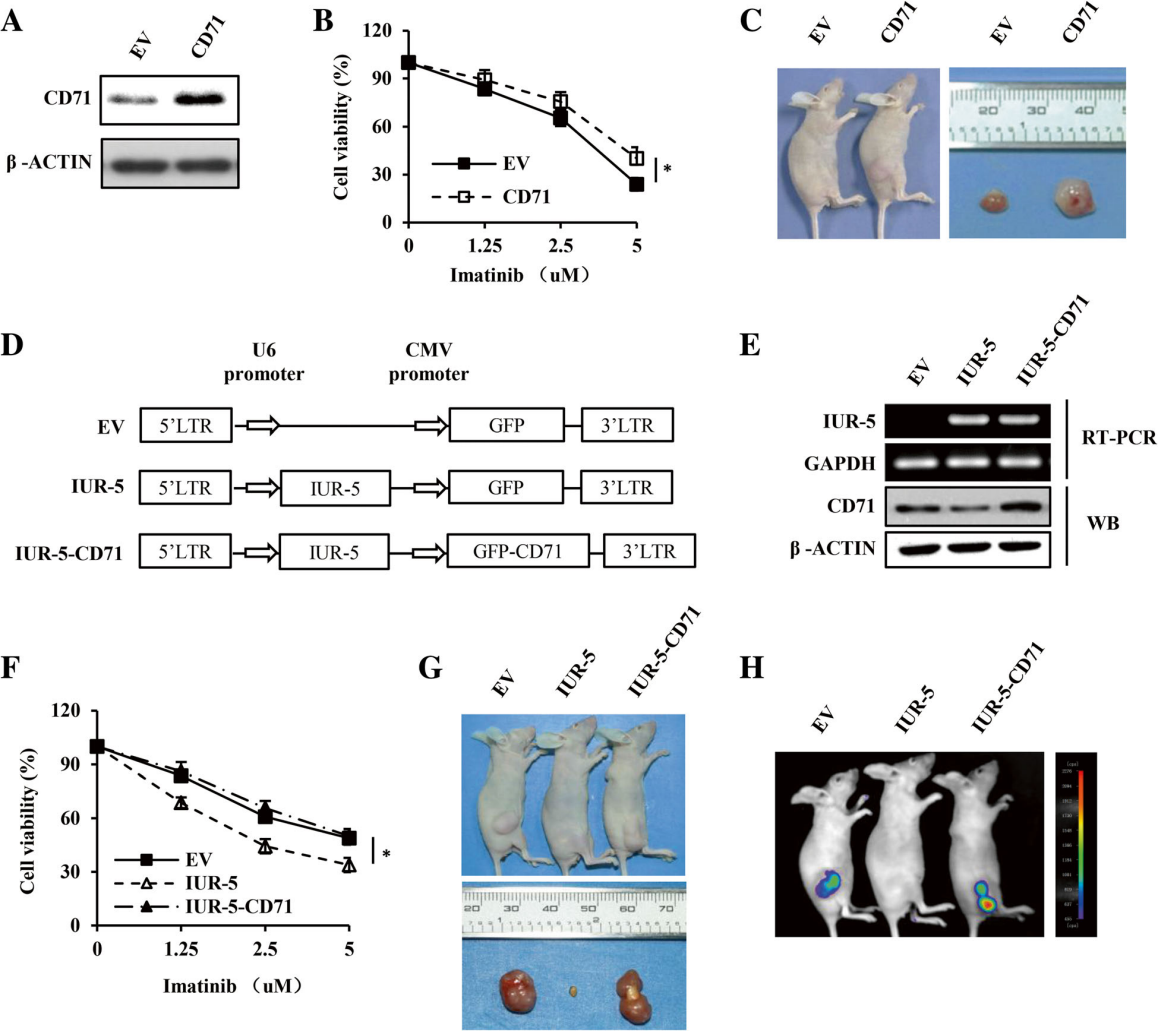
Overexpressing CD71 reverses the inhibitory effect of lncRNA-IUR-5 on cell survival and tumorigenesis of Abl transformants. **a**, Western blotting analysis of CD71 levels in K562 cell lines overexpressing CD71 and control. **b**, Cell viability of indicated K562 cell lines was analyzed by flow cytometry after treatment with indicated concentrations of imatinib for 36 h (n = 3; means ± SEM; *p < 0.05). **c**, Shown were representative images of nude mice subcutaneously injected with indicated K562 cell lines, and tumors were excised from these nude mice. **d**, Scheme of plasmids overexpressing lncRNA-IUR-5 alone (IUR-5), lncRNA-IUR-5 with CD71 (IUR-5-CD71), and EV. **e**, Western blotting and RT-PCR were performed to examine the protein and mRNA levels as indicated. **f**, Cell viability of indicated K562 cell lines were analyzed by flow cytometry after treatment with indicated concentrations of imatinib for 36 h (n = 3; means ± SEM; *p < 0.05). **g** and **h**, Shown were representative images of nude mice subcutaneously injected with indicated K562 cell lines. Tumors were excised from nude mice (**g**), and tumor growth was analyzed by bioluminescence imaging (**h**). Shown were representative images from at least three independent experiments with similar results

To further address the relationship between lncRNA-IUR-5 and CD71 in Abl transformants, we firstly examined the role of CD71 in Abl-transformed cell survival and tumorigenesis. For this, we generated K562 cell lines in which CD71 expression was altered (Fig. 7a and Additional file 2: Figure S5A). We observed that cell viability of K562 cell line overexpressing CD71 was significantly higher than that of control group upon treatment by imatinib (Fig. 7b). Moreover, the tumors induced by K562 cell line overexpressing CD71 were remarkably larger than that of control group (Fig. 7c). In contrast, cell viability and tumor growth of CD71 knockdown K562 cells were both significantly decreased compared with control groups (Additional file 2: Figure S5B-S5D). These results imply that CD71 can promote Abl-transformed cell survival and xenograft growth in nude mice.

We then investigated the ability of CD71 to reverse the inhibitory effect of lncRNA-IUR-5 on Abl-mediated tumorigenesis. We generated K562 cell lines overexpressing lncRNA-IUR-5 with or without CD71, and empty vector (Fig. 7d and e). As shown in Fig. 7f, cell viability of K562 cell line overexpressing lncRNA-IUR-5 alone was significantly lower than that of control cells, but forced expression of CD71 restored it to the level comparable to that of control. These data were consistently confirmed by tumor formation in the xenograft mouse model (Fig. 7g and h). Thus, our results demonstrate that forced expression of CD71 is able to reverse the inhibitory effect of lncRNA-IUR-5 on cell survival and tumorigenesis of Abl transformants, and suggest that lncRNA-IUR-5 inhibits Abl-mediated tumorigenesis through suppression of CD71.

## Discussion

Recently, increasing numbers of lncRNAs have been identified to be associated with cancers, which can be used as biomarkers for patient prognosis and potential therapeutic agents [35, 36]. Despite these progresses, the mechanisms underlying lncRNA function in Abl-induced transformation are poorly characterized. Here, we identified a conserved, imatinib-upregulated lncRNA-IUR, and demonstrated that lncRNA-IUR functioned as a suppressor to inhibit Abl-transformed cell survival in vitro and tumor growth in vivo. Importantly, murine lncRNA-IUR knockdown transgenic mice exhibited enhanced Bcr-Abl-mediated primary bone marrow transformation. Also, the inhibitory role of lncRNA-IUR in Abl-induced tumorigenesis was confirmed through in vivo leukemia transplant. Our results provide strong evidence that lncRNA-IUR may act as a critical tumor suppressor during Bcr-Abl-induced leukemogenesis. Moreover, we determined whether lncRNA-IUR suppressed Bcr-Abl-induced tumorigenesis through regulating cell cycle and cell viability. Our observations from in vitro experiments suggest that lncRNA-IUR is involved in the regulation of cell survival but not cell cycle progression of Abl transformants such as K562 and NS2 cells. However, the precise molecular mechanism remains to be further defined.

Abl oncoproteins induce tumorigenesis through complicated mechanisms, involving activation of signaling pathways that regulate cell survival and proliferation [2, 3, 5, 37]. Previous studies have shown that JAK2/STAT5 pathway was constitutively activated by Bcr-Abl in CML, resulting in uncontrolled cell survival and proliferation [2]. Bcr-Abl can cause tyrosine phosphorylation of suppressors of cytokine signaling 1 and 3 (SOCS-1 and SOCS-3), two potent suppressors of JAK2/STAT5 signaling [4]. In this study, we identified lncRNA-IUR-5 as a key negative regulator of STAT5-CD71 pathway in Abl transformants, as evidenced by profound inhibitory effects of lncRNA-IUR-5 overexpression on STAT5 activation and STAT5-mediated CD71 production. We observed that expression levels of lncRNA-IUR were low in Bcr-Abl-positive leukemic cells and v-Abl-positive mouse cell lines. Strikingly, treatment with the Abl kinase inhibitor imatinib resulted in a marked increase in lncRNA-IUR expression, suggesting that the expression of lncRNA-IUR is suppressed by Abl kinases-dependent mechanism in these cells, which may be required for efficient tumorigenesis induced by Abl oncogenes.

Furthermore, we demonstrated that the expression of lncRNA-IUR-5 was suppressed by Abl-dependent activation of STAT5. However, the mechanism by which activated STAT5 suppresses lncRNA-IUR-5 expression needs to be further investigated. In addition, the interaction between lncRNA-IUR-5 and STAT5 was found by RNA pull-down experiment and subsequent analysis. These data indicate that there exists reciprocal regulation between lncRNA-IUR-5 and STAT5 in Abl transformants. The exact domain of lncRNA-IUR-5 that is responsible for the interaction with STAT5 remains to be further determined.

It is worth noting that inhibition of STAT5 had no effect on the expression of lncRNA-IUR-6 and lncRNA-IUR-8, although our study demonstrated that inhibition of STAT5 upregulated the expression of lncRNA-IUR-5. These data imply that the regulatory mechanisms of lncRNA-IUR-6 and lncRNA-IUR-8 in Abl-mediated tumorigenesis were different from that of lncRNA-IUR-5, and might rely on other Abl kinase-activated signaling pathways. For this, we have conducted some preliminary studies. K562 cells were treated with LY294002 and AKTi respectively. Interestingly, inhibition of the PI3K/AKT/mTOR pathway only caused a significant upregulation of lncRNA-IUR-8 but not lncRNA-IUR-5 and lncRNA-IUR-6 (Additional file 2: Figure S6A-D). Thus, we speculate that the inhibitory effect of lncRNA-IUR-8 on Abl-mediated tumorigenesis may be dependent on the PI3K/AKT/mTOR pathway. To prove this, more experiments are on the way. Also, to unveil the regulatory mechanism of lncRNA-IUR-6 in Abl-mediated tumorigenesis, further research is warranted.

In this study, we also tested whether lncRNA-IUR-5 was regulated by STAT5 in other cell lines in the absence of Abl oncogenes. The results showed that knockdown of STAT5 upregulated the expression of lncRNA-IUR-5 in K562 cells (a positive control). However, knockdown of STAT5 did not affect the expression of lncRNA-IUR-5 in all cell lines tested, including 293 T, MCF-7, A549, and Huh7 cells (Additional file 2: Figure S6E). Thus, the results indicate that inhibitory effect of STAT5 on lncRNA-IUR-5 expression might be specific to Abl transformants rather than other cancer cells.

It has been shown that lncRNAs could regulate the expression of distal gene across different chromosomes (in trans) [38, 39], and the expression of adjacent gene on the same chromosome (in cis), especially for antisense lncRNAs [40, 41]. Since lncRNA-IUR is an antisense gene of SESN3 that is implicated in imatinib-inhibited PI3K/AKT/mTOR pathway [42, 43], we also examined if there was any functional relevance between lncRNA-IUR and SESN3 in Abl transformants. Our results showed that knockdown or overexpression of lncRNA-IUR did not obviously affect the mRNA (Additional file 2: Figure S7A and S7B) or protein level of SESN3 in K562 cells (Additional file 2: Figure S7C and S7D). On the other hand, knockdown or overexpression of SESN3 had no effect on lncRNA-IUR expression either (Additional file 2: Figure S7E and S7F). Hence, these findings suggest that there may be no regulatory relationship between lncRNA-IUR and SESN3 in Abl transformants.

In addition, we analyzed endogenous expression of lncRNA-IUR in Bcr-Abl-positive ALL patients and normal control. We found that lncRNA-IUR expression was remarkably low in leukemic cells derived from Bcr-Abl-positive ALL patients as compared with normal control. Likely, the inhibitory function of lncRNA-IUR was restricted in Bcr-Abl-positive ALL patients due to its impaired expression by unknown mechanism, leading to development of Bcr-Abl-induced leukemogenesis. However, our number of Bcr-Abl-positive clinical samples was limited, and a large-scale study is ongoing to validate the reliability of our results. Additionally, patient-derived xenografts (PDXs) that surgically transferring clinical tumor samples into immunodeficient mice are expected to be better research strategies for precision cancer medicine to deal with tumor cellular heterogeneity [44]. Given the fact that Bcr-Abl-mediated leukemogenesis is a heterogeneous disease, and some leukemia cells are resistant to imatinib and other TKIs, it is worth utilizing PDXs model to study and develop precisely targeted therapies for imatinib-resistant leukemia with integration of clinical data.

## Conclusions

In summary, this study identified lncRNA-IUR family as a critical suppressor in Abl-mediated tumorigenesis. We demonstrated that lncRNA-IUR inhibited Abl transformed cell survival in vitro and tumor growth in vivo. Furthermore, the results revealed that lncRNA-IUR-5 impeded Abl-mediated tumorigenesis through suppression of the STAT5-CD71 pathway. The findings provide novel insights into the functional involvement of lncRNAs in leukemogenesis.

## Additional files

Additional file 1: Supplementary materials and methods. (DOCX 19 kb)

Additional file 2: **Figure S1**. LncRNA-IUR is a conserved, imatinib-upregulated lncRNA family, related to Fig. 1. **Figure S2**. Analysis of functional relevance of lncRNA-IUR to Abl transformant survival and tumorigenesis in a xenograft mouse model, related to Figs. 2 and 3. **Figure S3**. Silencing murine lncRNA-IUR in transgenic mice promotes Abl-induced leukemia development in vivo, related to Figs. 4 and 5. **Figure S4**. Identification of binding protein(s) with lncRNA-IUR-5, related to Fig. 6. **Figure S5**. Disruption of CD71 decreases K562 cell survival and xenograft growth in nude mice, related to Fig. 7. **Figure S6**. Analysis of lncRNA-IUR expression under inhibition of PI3K/AKT/mTOR pathway or STAT5 activity in indicated cell lines, related to Fig. 6. **Figure S7**. LncRNA-IUR does not affect the mRNA and protein level of SESN3 in K562 cells. **Table S1.** The Target Sequences of shRNAs. **Table S2.** Sequences of Primers Used in This Study. (DOCX 15300 kb)

## Abbreviations

ALL: acute lymphoblastic leukemia
BMCs: bone marrow cells
ceRNA: competitive endogenous RNA
CML: chronic myeloid leukemia
CPC: coding potential calculator
CTCs: circulating tumor cells
HE: hematoxylin and eosin
IUR: imatinib-upregulated lncRNA
JAK/STAT: janus family of kinase/signal transducer and activator of transcription
LncRNAs: Long noncoding RNAs
ORF: open reading frame
PBCs: peripheral blood cells
PDXs: patient-derived xenografts
PI3K/AKT: phosphatidylinositide 3-kinase/protein kinase B
PLT: platelet
PTEN: phosphatase and tensin homolog
RBCs: red blood cells
RIP: RNA immunoprecipitation
shRNA: short hairpin RNA
SOCS: suppressors of cytokine signaling
TfR: transferrin receptor protein
TKIs: tyrosine kinase inhibitors
WBCs: white blood cells
