# Supplementary material for: Novel lncRNA-IUR suppresses Bcr-Abl-induced tumorigenesis through regulation of STAT5-CD71 pathway
Source: Mol Cancer. 2019 Apr 8;18:84. doi: 10.1186/s12943-019-1013-3 (PMC6454664; doi:10.1186/s12943-019-1013-3)
Supplement: Supplementary file 1 — Supplementary materials and methods. (DOCX 19 kb) [file 12943_2019_1013_MOESM1_ESM.docx]

**Supplementary Materials and Methods**

**Antibodies and Reagents**

The following antibodies were used in this study: anti-FLAG (F1804 M2, Sigma), anti-STAT5 (9358 3H7, Cell Signaling), anti-phospho-STAT5 (Tyr694) (9359 C11C5, Cell Signaling), anti-Transferrin receptor/CD71 (10084-2-ap, Proteintech), and anti-SESN3 (11431-2-ap, Proteintech). The inhibitors were purchased as follows: Imatinib (SC-267106, Santa Cruz), STAT5-IN-1 (HY-101853, MedChemExpress), LY294002 (L9908, Sigma), and AKTi (124018, Merck). All other antibodies and reagents were obtained as previously described (Guo et al. 2015, Guo et al. 2014, Yang et al. 2013).

**DNA Construction and Cell Line Generation**

SESN3, CD71 and each transcript of lncRNA-IUR were respectively subcloned into pNL-EGFP-CMV-WPREDU3 vector, in which taking place of EGFP, to generate pNL-SESN3, pNL-CD71, and pNL-lncRNA-IUR. Bcr-Abl was subcloned into pMIG-IRES-GFP to generate pMIG-Bcr-Abl-GFP. LncRNA-IUR-5 and CD71 were cloned into pll3.7 vector [simultaneously](javascript:;).

Gene overexpression cell lines were generated by infecting the cells with viruses encoding indicated gene using the viral vector pNL or pll3.7 as previously described. ShRNA-expressing stable cell lines were generated by infection of the cells with lentiviruses expressing specific shRNAs in pSIH-H1-GFP vector (System Biosciences) as described previously. The shRNA target sequences are listed in Additional file 1: Table S1.

**Subcellular Fractionation**

Cytoplasmic and nuclear fractions were separated as described previously (Yoon et al., 2012). Briefly, K562 cells were lysed with TD buffer (25 mM Tris-HCl pH 7.4; 100 mM NaCl; 5 mM KCl; and 0.7mM Na2HPO4) containing 1% NP-40 for 5 min. The lysates were centrifuged with 12,000 rpm for 5 min at 4ºC. The supernatant was collected and used for the cytoplasmic fraction. The nuclear pellets were washed with TD buffer containing 0.5% NP-40 on ice for 5 min and collected after centrifugation at 4ºC for 5 min at 12,000 rpm.

**RT-PCR, Quantitative Real-time PCR, and Western Blotting**

Total RNA was extracted from cells or tissues using TRIzol reagent (Invitrogen, Carlsbad, CA, USA). The cDNA was synthesized using 5 ug of total RNA, reverse transcriptase (RT; Promega, Madison, WI, USA), and oligo (dT) primers (Takara, Dalian, P.R. China). The following PCR was performed using rTaq DNA polymerase and quantitative PCR using SYBR PremixEx TaqII (TaKaRa, Tokyo, Japan) with gene specific primers shown in Additional file 1: Table S2. GAPDH was chosen as a reference gene for internal standardization.

For Western blotting, samples were separated on SDS-polyacrylamide gel, transferred to a nitrocellulose membrane, and probed with antibodies as indicated.

**Histopathological Analysis**

Mouse spleens were fixed in 4% paraformaldehyde and then embedded in paraffin. 4 mm thick sections were prepared and stained with hematoxylin and eosin (HE). The slides were visualized under an Olympus BH-2 microscope (Tokyo, Japan).

**Cell Cycle Analysis**

Cell cycle analysis was performed as previously described. Briefly, cells were collected and fixed in 75% ethanol at 4°C overnight, then washed and incubated with propidium iodide (5 mg/ml with 0.1% RNase A) for 30 min. The samples were analyzed with a fluorescence-activated cell sorter (BD Bioscience).

**RNA-seq Analysis**

Total RNAs were isolated from three independent groups of K562 cell lines expressing shRNA targeting lncRNA-IUR-5 and control cells, using TRIzol reagent (Invitrogen, Carlsbad, CA, USA). RNA libraries were prepared for sequencing using standard Illumina protocols. At the data procession step, Illumina Casava software was used for basecalling. Sequenced reads were trimmed for adaptor sequence, and masked for low-complexity or low-quality sequence, then mapped to hg38 whole genome. Reads Per Kilo bases per Million reads (RPKM) were calculated and analyzed using samtools v0.1.19.

**RNA Pull-down Assay**

Briefly, K562 cell lines overexpressing S1, lncRNA-IUR-5, lncRNA-IUR-5-S1 were generated as previously described. Then, these cell lines were lysed in ice-cold gentle lysis buffer (GLB), and followed by incubation with streptavidin beads (Invitrogen, 65601) at 4°C for 3 h. After washing with ice-cold GLB five times, the purified ribonucleoproteins (RNPs) were eluted from the beads in 2X SDS sample buffer by heating at 100°C for 5 min, and resolved by 10% SDS-PAGE. The proteins in the gel were excised and followed by mass spectrometry.
