# Supplementary material for: Novel lncRNA-IUR suppresses Bcr-Abl-induced tumorigenesis through regulation of STAT5-CD71 pathway
Source: Mol Cancer. 2019 Apr 8;18:84. doi: 10.1186/s12943-019-1013-3 (PMC6454664; doi:10.1186/s12943-019-1013-3)
Supplement: Supplementary file 2 — Figure S1. LncRNA-IUR is a conserved, imatinib-upregulated lncRNA family, related to Fig. 1. Figure S2. Analysis of functional relevance of lncRNA-IUR to Abl transformant survival and tumorigenesis in a xenograft mouse model, related to Figs. 2 and 3. Figure S3. Silencing murine lncRNA-IUR in transgenic mice promotes Abl-induced leukemia development in vivo, related to Figs. 4 and 5. Figure S4. Identification of binding protein(s) with lncRNA-IUR-5, related to Fig. 6. Figure S5. Disruption of CD71 decreases K562 cell survival and xenograft growth in nude mice, related to Fig. 7. Figure S6. Analysis of lncRNA-IUR expression under inhibition of PI3K/AKT/mTOR pathway or STAT5 activity in indicated cell lines, related to Fig. 6. Figure S7. LncRNA-IUR does not affect the mRNA and protein level of SESN3 in K562 cells. Table S1. The Target Sequences of shRNAs. Table S2. Sequences of Primers Used in This Study. (DOCX 15300 kb) [file 12943_2019_1013_MOESM2_ESM.docx]

**Additional file 2**

**
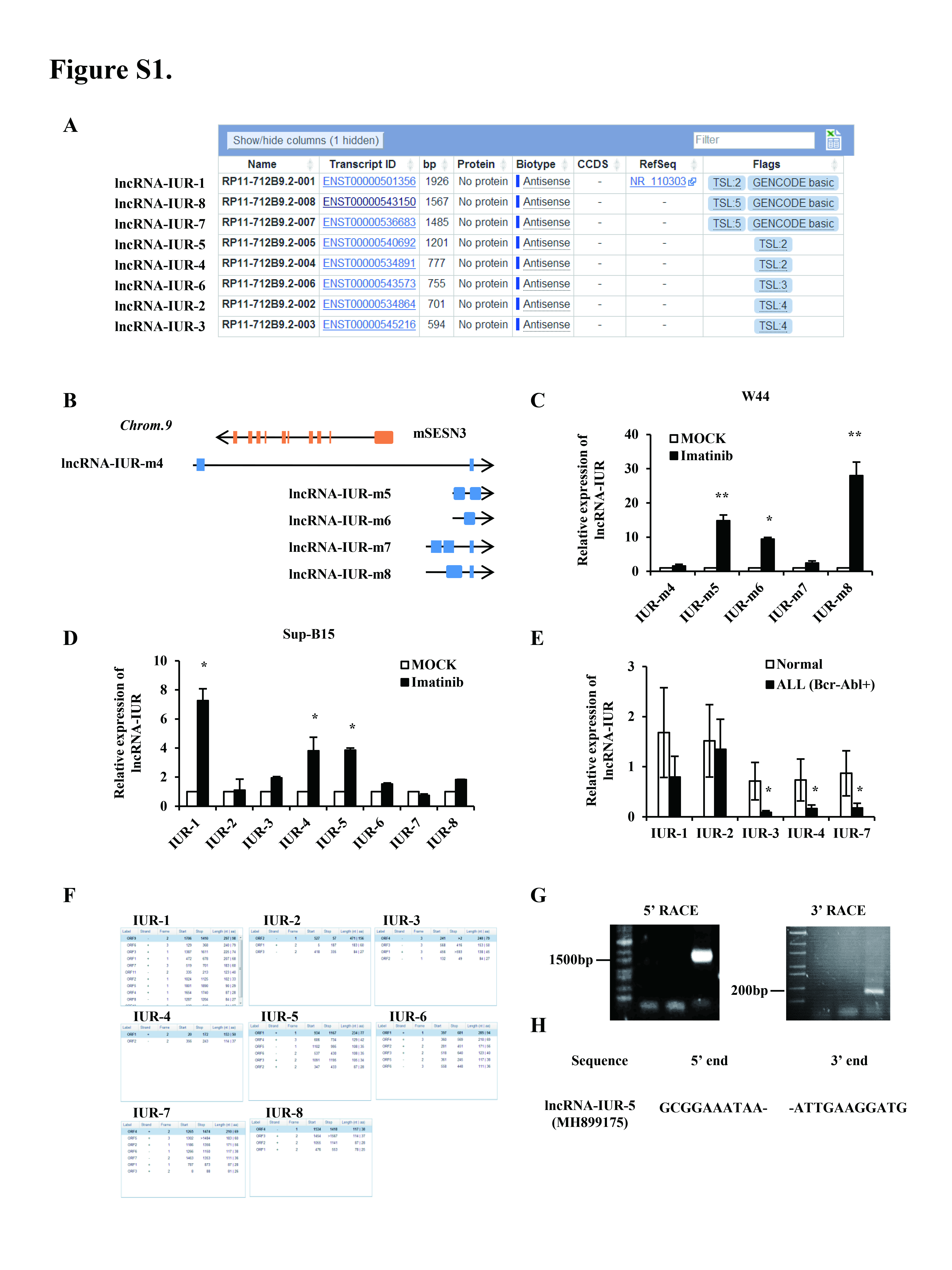
**

**Figure S1. LncRNA-IUR is a conserved, imatinib-upregulated lncRNA family, related to Fig. 1.** **A,** Information of lncRNA-IUR family from Ensembl website (<http://asia.ensembl.org/index.html>). **B,** The paradigm of the mouse genomic location of lncRNA-IUR family (blue) and mSESN3 (orange). The orientation of arrows indicated the transcription direction. **C** and **D,** Quantitative real-time PCR analysis of lncRNA-IUR expression in W44 (C) and Sup-B15 (D) cells upon imatinib treatment (n = 3; means ± SEM; **p* < 0.05; ***p* < 0.01). **E,** Quantitative real-time PCR analysis of lncRNA-IUR expression in primary leukemic cells from Bcr-Abl-positive ALL patients (n = 5; means ± SEM; **p* < 0.05). **F,** The potential ORFs analysis for each transcript of lncRNA-IUR by Open Reading Frame Finder (<https://www.ncbi.nlm.nih.gov/orffinder/>). **G,** Agarose gel analysis of 5’ and 3’ RACE PCR products of lncRNA-IUR-5. **H,** Shown were the 5 end and 3 end sequence of lncRNA-IUR-5.

**
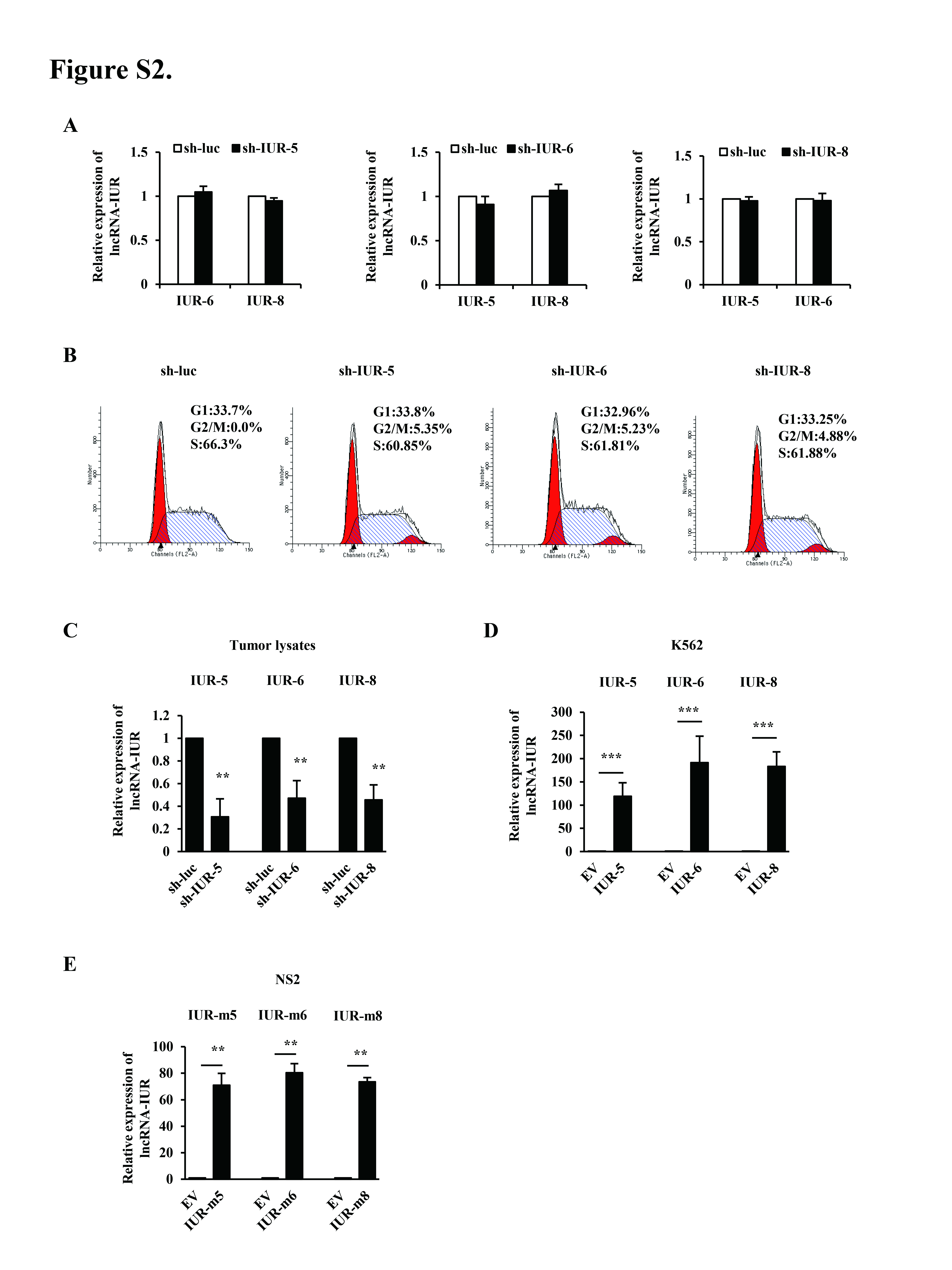
**

**Figure S2.** **Analysis of functional relevance of lncRNA-IUR to Abl transformant survival and tumorigenesis in a xenograft mouse model, related to Fig. 2 and 3.** **A,** Quantitative real-time PCR was performed to examine lncRNA-IUR expression in K562 cell lines stably expression shRNA target lncRNA-IUR-5, -6, or -8 (n = 3; means ± SEM). **B,** Cell cycle progression was analyzed by flow cytometry in indicated cells. **C,** LncRNA-IUR expression in tumors excised from nude mice injected with lncRNA-IUR knockdown K562 cell lines were analyzed by quantitative real-time PCR (n = 3; means ± SEM; ***p* < 0.01). **D,** Quantitative real-time PCR was performed to examine lncRNA-IUR expression in K562 cell lines overexpressing lncRNA-IUR-5, -6, -8 or empty vector (EV) (n = 3; means ± SEM; ****p* < 0.001). **E,** Quantitative real-time PCR was performed to examine lncRNA-IUR expression in NS2 cell lines overexpressing lncRNA-IUR-m5, -m6,-m8 or EV (n = 3; means ± SEM; ***p* < 0.01).

**
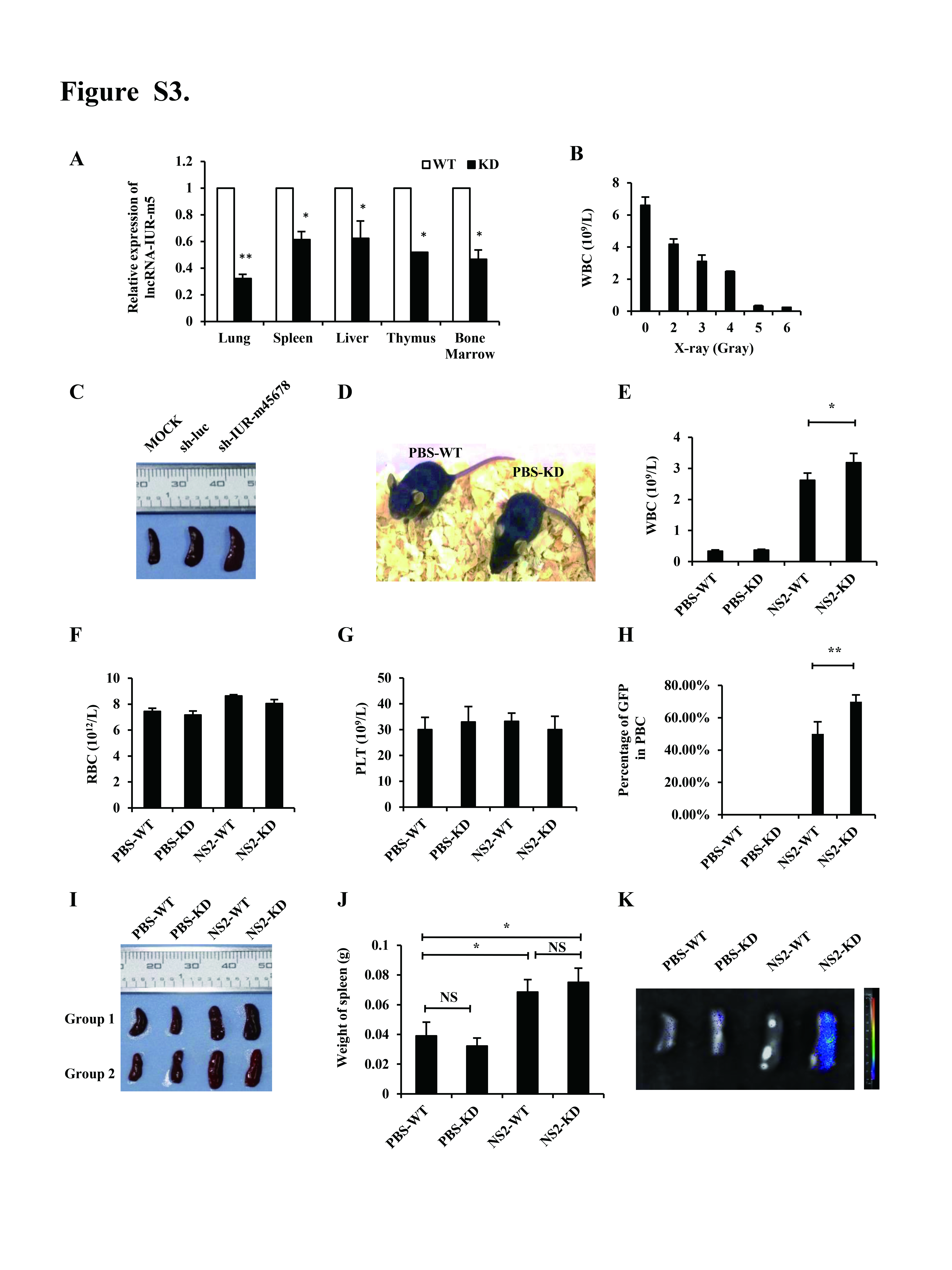
**

**Figure S3. Silencing murine lncRNA-IUR in transgenic mice promotes Abl-induced leukemia development *in vivo*, related to Fig. 4 and 5.** **A,** Quantitative real-time PCR was performed to examine the lncRNA-IUR-m5 expression in multiple organs of lncRNA-IUR KD transgenic mice (n = 5; means ± SEM; **p* < 0.05; ***p* < 0.01). **B,** Quantity of WBCs in peripheral blood of C57BL/6J mice were measured at the 15^th^ days after X-ray irradiation under different intensity. **C,** Shown were representative images of spleens from indicated mice at the 8^th^ day after *in vivo* leukemia transplantation. **D,** Representative photo shown PBS-KD and PBS-WT mice at the 8^th^ day after *in vivo* leukemia transplantation. **E-G,** Quantity of WBCs (E), RBCs (F) and PLTs (G) in peripheral blood of indicated mice were detected by blood routine examination (n = 8; means ± SEM; **p* < 0.05). **H,** Percentage of GFP-positive NS2 cells in PBCs of indicated mice were detected by flow cytometry (n = 5; means ± SEM; ***p* < 0.01). **I,** Representative photo of two groups of spleens excised from indicated mice. **J,** Weight of spleens from indicated mice were measured at the 15^th^ days after *in vivo* transplant (n = 8; means ± SEM; **p* < 0.05; ns = no significance). **K,** Bioluminescent imaging was performed to examine distribution of GFP-positive NS2 cells in spleens of indicated mice at the 8^th^ day after *in vivo* leukemia transplantation. Shown were representative images from at least three independent experiments with similar results.

**
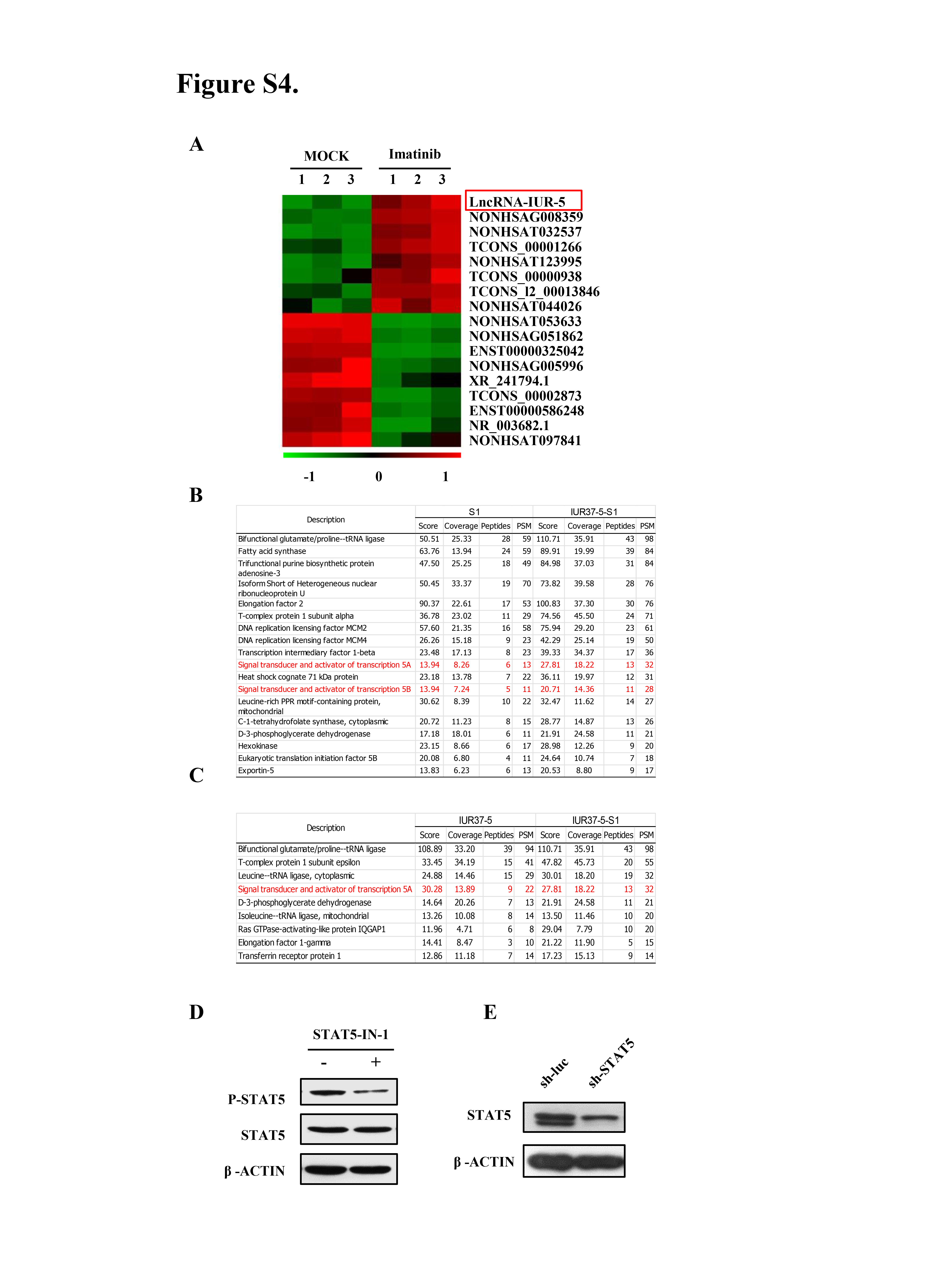
**

**Figure S4. Identification of binding protein(s) with lncRNA-IUR-5, related to Fig. 6.** **A,** Shown were representative lncRNAs differentially expressed in K562 cells treated with or without imatinib. **B** and **C,** Potential binding protein(s) of lncRNA-IUR-5 were pulled-down and identified by mass spectrometry. STAT5A and STAT5B were found through comparing sample S1 with IUR-5-S1 (B). STAT5A were found through comparing sample IUR-5 with IUR-5-S1 (C). **D,** Phosphorylation of STAT5 in K562 cells upon STAT5-IN-1 (50 μM, 6 h) treatment was analyzed by Western blotting. **E,** Protein level of STAT5 in K562 cell lines stably expressing sh-STAT5 or sh-luc was analyzed by Western blotting.

**
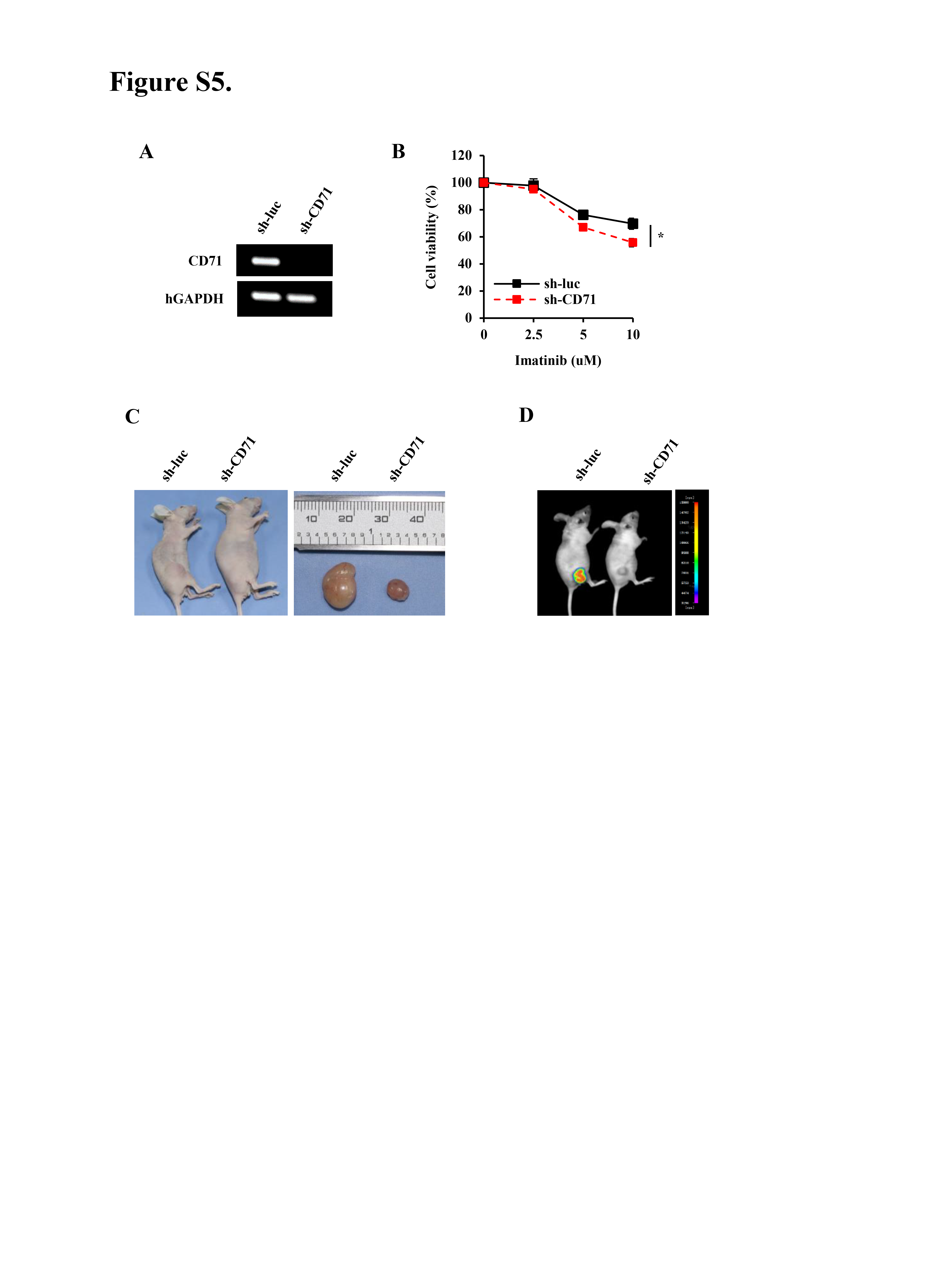
**

**Figure S5. Disruption of CD71 decreases K562 cell survival and xenograft growth in nude mice,** **related to Fig. 7.** **A,** RT-PCR was performed to examine CD71 expression in K562 cell lines stable expressing sh-CD71 or sh-luc. **B,** Cell viability of indicated K562 cell lines was analyzed by flow cytometry after treatment with indicated concentrations of imatinib for 36 h (n = 3; means ± SEM; **p* < 0.05). **C** and **D,** Nude mice were subcutaneously injected with K562 cell lines stable expressing sh-CD71 or sh-luc. Tumors were excised from indicated mice (C), and tumor growth was measured by bioluminescent imaging (D). Shown were representative images from at least three independent experiments with similar results.

**
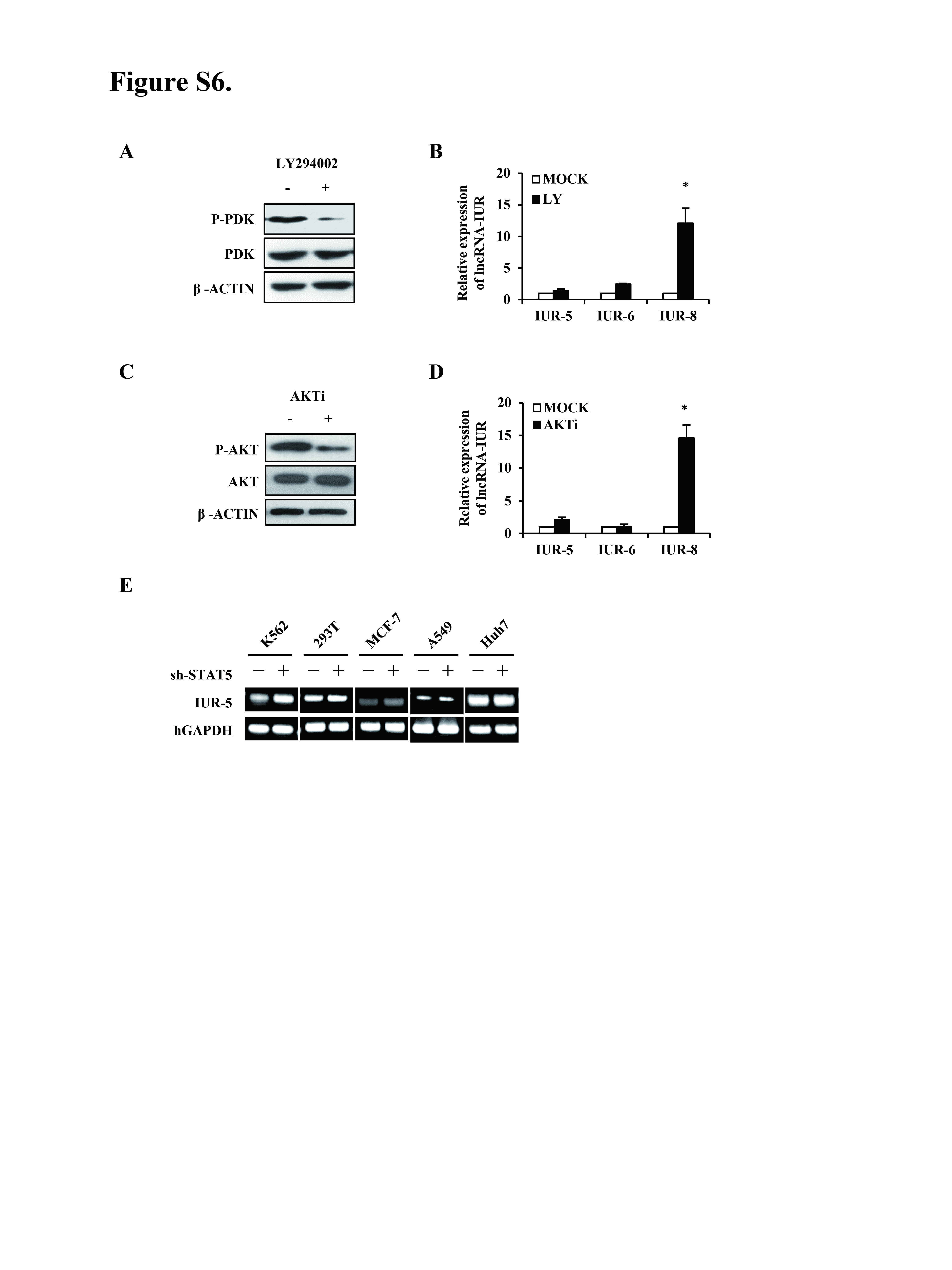
**

**Figure S6. Analysis of lncRNA-IUR expression under inhibition of PI3K/AKT/mTOR pathway or STAT5 activity in indicated cell lines, related to Fig. 6.** **A** and **B,** Phosphorylation of PDK and lncRNA-IUR expression in K562 cells upon LY294002 (5 μM, 6 h) treatment were analyzed by Western blotting (A) and quantitative real-time PCR (B) (n = 3; means ± SEM; **p* < 0.05). **C** and **D,** Analysis of AKT phosphorylation and lncRNA-IUR expression in K562 cells after AKTi (5 μM, 6 h) treatment were performed as described in A and B (n = 3; means ± SEM; **p* < 0.05). **E,** LncRNA-IUR-5 expression in indicated human cancer cells stable expressing sh-STAT5 or sh-luc was examined by RT-PCR.

**
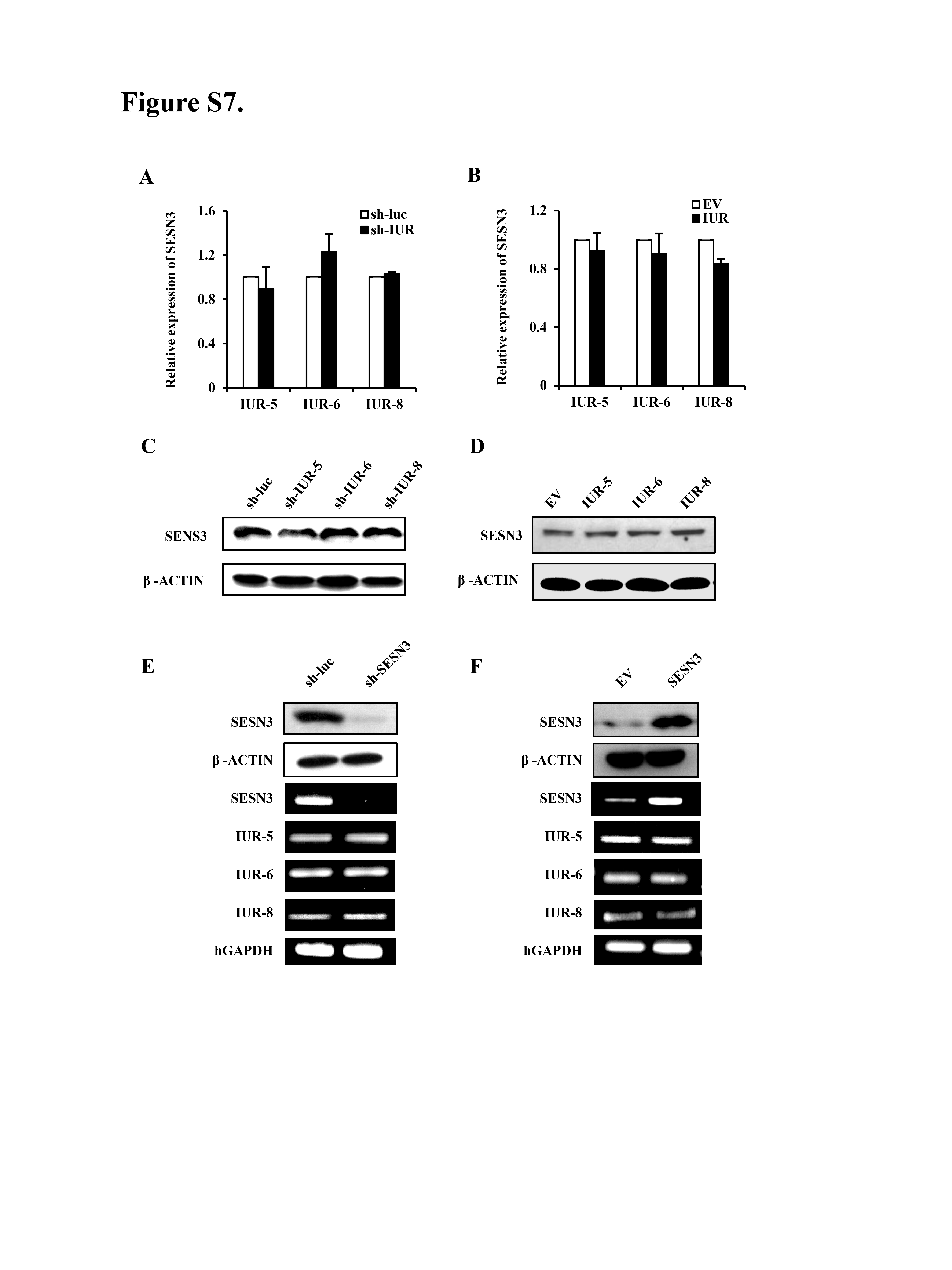
**

**Figure S7. LncRNA-IUR does not affect the mRNA and protein level of SESN3 in K562 cells.** **A** and **B,** The mRNA level of SESN3 in lncRNA-IUR knockdown K562 cell lines (A) or lncRNA-IUR overexpressing K562 cell lines (B) was examined by quantitative real-time PCR. **C** and **D,** The protein level of SESN3 in indicated K562 cell lines was analyzed by Western blotting. **E** and **F,** Western blotting and RT–PCR were performed to examine the protein and mRNA levels as indicated.

| **Table S1. The Target Sequences of shRNAs** | |
| --- | --- |
| **Name** | T**arget sequences** |
| sh-IUR-5 | GCTAGTATCAGGTTTAAATAG |
| sh-IUR-6 | GTCTCCAGCGTACCAGGATTT |
| sh-IUR-8 | GCTACGCCCCCTTCTCTAGGT |
| sh-IUR-m45678 | GGCCTGCAATTTGTAGAATGT |
| sh-SESN3 | GCGTTTGTGATCTTGCTAATG |
| sh-CD71 | GCTCTAGCTACTTGGACTATT |
| sh-STAT5 | GCAGCAGACCATCATCCTG |

| **Table S2. Sequences of Primers Used in This Study** | | |
| --- | --- | --- |
| **Genes** | **Sequences** | |
|  | **Forward** | **Reverse** |
| IUR-1 | CTTCCGAGAGCCCCACAGTCCT | CACCACACCCAGCAAATTACATAATC |
| IUR-2 | TTGCCTGAAGACTGGGAAGTAAGAG | CAAGACTTTCCAGAAAATGAGCTTG |
| IUR-3 | GTAAGAGTAGGGGCGTCGGAGGA | GAGCTGGTGCTTCTAACCACAGTGC |
| IUR-4 | GCCAGGACTACAGAGCAG | CGATCTTTGTTAGAGGGTG |
| IUR-5 | TTGGGACAAACCTACCTC | GCATAACACCACCCTGAA |
| IUR-6 | CAGCGTACCAGGATTTGT | GGTTTGAAATAAAATTCCCT |
| IUR-7 | CAAGGCAGAAGCAACCGAATA | TCCCTTCAGTTCCGAGCC |
| IUR-8 | CTCTGGTTTAGCGGTTTCC | CCTTTAGCGATCTTTGTTAG |
| IUR-m4 | GGTTTCTCTTTCCTCCCCTTC | GTTCTTTAAAACCATCTCCACAG |
| IUR-m5 | CCGACCCACTGGTTTACT | TACCCAAATCACTGCTGTTC |
| IUR-m6 | GTTAGCATAACAAAGCCATTAG | ACCCAGCATCAAACCACAC |
| IUR-m7 | CTTCATGGTGCCTTTCGG | ACCTTTGGCGTGGCTTG |
| IUR-m8 | TGGGTTAGTTAGGTAGTGGGG | CAGCCGTAGGTAGTTGGTC |
| SESN3  (human) | TCTCCCTGGTGAACAGAC | GGTCAAATGCCGAGTTAT |
| CD71  (human) | CAGTTTCCACCATCTCGGTCATCA | AAGGTATCCCTCTAGCCATTCAGT |
| hGAPDH  (human) | AGAAGGCTGGGGCTCATTTG | AGGGGCCATCCACAGTCTTC |
| mGAPDH  (mouse) | GCCTCGTCCCGTAGACAAAA | CCCTTTTGGCTCCACCCTTC |
